# What Cognitive Mechanism, When, Where, and Why? Exploring the Decision Making of University and Professional Rugby Union Players During Competitive Matches

**DOI:** 10.3389/fpsyg.2021.609127

**Published:** 2021-05-12

**Authors:** Michael Ashford, Andrew Abraham, Jamie Poolton

**Affiliations:** ^1^Faculty of Health and Life Sciences, Coventry University, Coventry, United Kingdom; ^2^Research Centre for Sport Coaching, Carnegie School of Sport, Leeds Beckett University, Leeds, United Kingdom

**Keywords:** rugby union, decision making, time, mental representation, situation, self-confrontation

## Abstract

Over the past 50 years decision making research in team invasion sport has been dominated by three research perspectives, *information processing*, *ecological dynamics*, and *naturalistic decision making*. Recently, attempts have been made to integrate perspectives, as conceptual similarities demonstrate the decision making process as an interaction between a players perception of game information and the individual and collective capability to act on it. Despite this, no common ground has been found regarding what connects perception and action during performance. The differences between perspectives rest on the role of stored mental representations, that may, or may not facilitate the retrieval of appropriate responses in time pressured competitive environments. Additionally, in team invasion sports like rugby union, the time available to players to perceive, access memory and act, alters rapidly between specific game situations. Therefore, the aim of this study was to examine theoretical differences and the mechanisms that underpin them, through the vehicle of rugby union. Sixteen semi-elite rugby union players took part in two post-game procedures to explore the following research objectives; (i) to consider how game situations influence players perception of information; (ii) to consider how game situations influence the application of cognitive mechanisms whilst making decisions; and (iii) to identify the influence of tactics and/or strategy on player decision making. Deductive content analysis and elementary units of meaning derived from self-confrontation elicitation interviews indicate that specific game situations such as; the lineout, scrum or open phases of play or the tackle situation in attack or defence all provide players with varying complexity of perceptual information, formed through game information and time available to make decisions. As time increased, players were more likely to engage with task-specific declarative knowledge-of the game, stored as mental representations. As time diminished, players tended to diagnose and update their knowledge-in the game in a rapid fashion. Occasionally, when players described having no time, they verbalised reacting on instinct through a direct connection between perception and action. From these findings, clear practical implications and directions for future research and dissemination are discussed.

## Introduction

At all levels of participation, the game of rugby union is an intense physical contest, where frequent high-speed collisions and forceful contacts define each phase of the game (Garraway et al., 2000; Reardon et al., 2017; Tee et al., 2018). The increasing physical demands of the sport that have come with its professionalisation, has been matched by a focus on developing the physical robustness of all players (Cunniffe et al., 2009; Austin et al., 2011; Tee et al., 2017). Consequently, where physical markers may have previously differentiated players, the gains in this area are now becoming more marginal (O’Connor and Larkin, 2015). A more consistent differentiator of performance in invasion sports can be aligned with expertise in game intelligence and decision making (Farrow and Raab, 2008; Memmert, 2011; O’Connor and Larkin, 2015; Gleeson and Kelly, 2020). This work shows that players, both individually and collectively, must be able to consistently select the appropriate courses of action to meet their intended goal, to score more points than their opponent (Richards et al., 2012, 2017). Given these consistent findings, supporting a coach’s understanding of and role in developing this skill set seems crucial (Bar-Eli et al., 2011).

**FIGURE 1:**
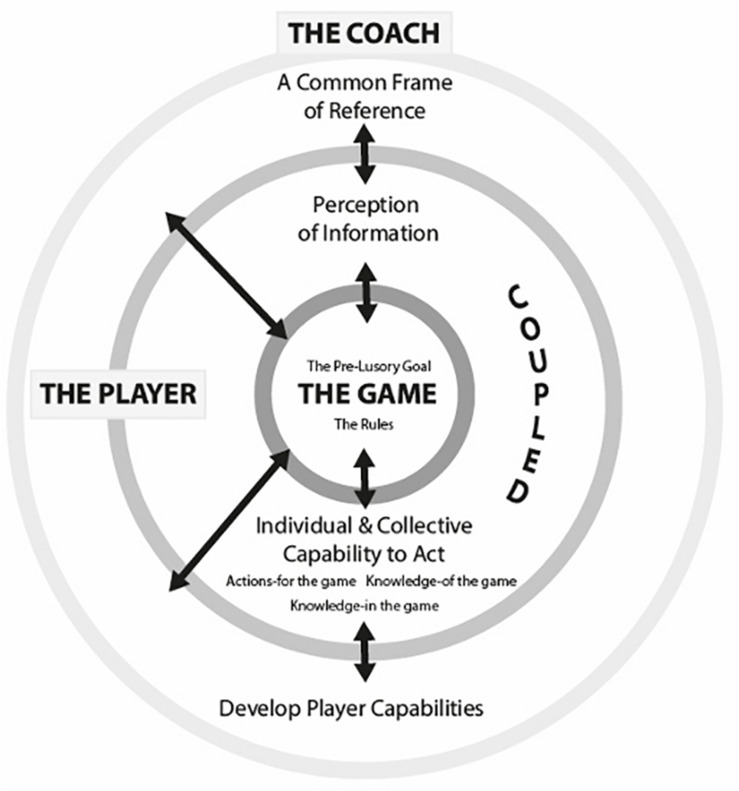
A unified conceptual framework for player decision making in invasion team sports (Ashford et al., 2021).

The significance of player decision making in invasion sports is emphasised by the ever-increasing volume of research dedicated to making sense of it (Toner et al., 2015; Araújo et al., 2019; Raab and Araújo, 2019; Williams and Jackson, 2019; Gleeson and Kelly, 2020). Currently, there is no agreed perspective on how players make decisions or, in turn, how coaches can best develop their players decision making (Araújo et al., 2019; Raab and Araújo, 2019; Williams and Jackson, 2019). Instead, three distinct views have emerged that offer different interpretations of the role of cognitive mechanisms within a players decision making process (Gigerenzer and Selten, 2002; Raab, 2003). Predominately, these differences centre on the presence or absence of memory representations that facilitate the selection of a course of action (Cappuccio, 2015; Raab and Araújo, 2019). The *information processing* view is that decisions are made through a deliberate and conscious interaction with memory representations, which have been formed over time (Toner and Moran, 2015; Birch et al., 2019). Conversely, the *ecological* view is that decision making occurs through a direct connection between a player and their environment, without reference to memory representation (Cappuccio, 2015; Raab and Araújo, 2019). Finally, the *naturalistic decision making* view is that conscious recourse to memory representations is dependent on the typicality of the information presented to the decision maker (Klein et al., 1986, 2010); typical situations reduce reference to memory representations (Macquet, 2009; Macquet and Kragba, 2015; Johnston and Morrison, 2016). The divergency in viewpoint has catalysed authors to explain findings in different ways and adopt distinct terminology to capture similar concepts. The question of language is also confounded by conflicting practical implications offered across perspectives (Richards and Collins, 2020). Unfortunately, coaches are left to navigate through convoluted solutions to an intricate practical problem. To temper these differences, Ashford et al. (2021) developed a communal language, housed in a unified conceptual framework, which attempts to facilitate the assimilation of knowledge borne from each perspective and to progress collective understanding of player decision making (Figure 1).

The unified conceptual framework compartmentalises decision making into three components: the *game*; the *player*; and the *coach*. None are mutually exclusive, as they interact with the other (Passos et al., 2008; Richards et al., 2017). The *Game* sits at the centre, as the goal and rules of the game interact to create problems that both the *Player* and the *Coach* need to solve. The player interacts directly with both the game and the coach, by performing shared solutions to the problems the game presents (Ashford et al., 2021). The Coach acts as a central agent in (co)creating, sharing and developing a view of the game with players through on and off field planning and delivery of coaching.

For players, the decision making process is actuated by the perception of *The Game* (Correia et al., 2012; Johnston and Morrison, 2016). *Rules* create the landscape of contextualised game information that players, in their quest to achieve the *Pre-Lusory goal* (i.e., to score more points than your opponent; Suits, 1978), are individually and collectively required to act on (Correia et al., 2012; Macquet and Kragba, 2015; Johnston and Morrison, 2016; Birch et al., 2019). Furthermore, the *rules* constrain players to select from a finite list of legal actions (Ashford et al., 2020).

Through interaction with the game, decision making of *The Player* is, in part, governed through task dependent *Perception of Information* (Ashford et al., 2021). Research has demonstrated that skilled perception is task dependent, that is, player’s visual fixations and verbal descriptions change in line with changes in the demands of the task, presumably as they search for means to a successful outcome (Roca et al., 2011, 2012, 2013). Moreover, expert players demonstrate an improved connection to the information offered by the performance environment over time (Abernethy et al., 2008; Esteves et al., 2012; Araújo et al., 2019; Williams and Jackson, 2019). Players’ perception of game information is inextricably linked with an *Individual and Collective Capability to Act* (Silva et al., 2014; Richards et al., 2017); more specifically, players *Actions-for the Game*, *Knowledge-of the Game* and *Knowledge-in the Game*. *Actions-for the Game* are shaped by the physical (e.g., speed) (Passos et al., 2012) and technical (Paterson et al., 2013) attributes of players. With experience, game information and relevant capabilities become coupled and refined, as inherent understanding of action capabilities more effectively guides the player to highly salient game information (Jackson et al., 2006; Davids et al., 2008). *Knowledge-of the Game* connects the search for game information and the selection of a relevant capability to act together through a deeper understanding of why specific decisions are more appropriate in one situation than another (McPherson and Vickers, 2004; Poplu et al., 2008; Lex et al., 2015; Johnston and Morrison, 2016). This characteristic of expertise has been attributed to the development over time of task-specific declarative knowledge stored as verbal and/or visual memory representations (Birch et al., 2019). Additionally, it has been proposed that players are required to update their *knowledge-in the game* in order to adapt to changes in expectations (Macquet and Kragba, 2015). So, when new information arises, such as an opposing player showing unexpected behaviour, player’s situation-specific knowledge requires updating (McPherson and Vickers, 2004).

Finally, within the framework, the *Coach* is responsible for creating a *Common Frame of Reference* to facilitate the collective response of their players to the game (Tee et al., 2018; Ashford et al., 2021). There is a general consensus across perspectives that the coordination of team play is a product of reference to a *global top-down* shared mental model (Richards et al., 2012, 2017) and the *local bottom-up* self-organisation of players through a shared perception and response to game information (Steiner et al., 2017; Ribeiro et al., 2019a, b), which the coach can influence by developing their *players’ capabilities*. However, at this point, this common ground remains conceptual (Steiner et al., 2017; Ribeiro et al., 2019a; Richards and Collins, 2020) as empirical research has been reduced to the study of top-down (Richards et al., 2012) and bottom-up (Bourbousson et al., 2010, 2011) approaches alone (Richards and Collins, 2020). In response, the current study investigates when, how and why players adopt top-down (shared mental model) or bottom-up (shared affordances) decision making processes (Richards and Collins, 2020).

In developing the unified conceptual framework Ashford et al. (2021) created a view on the, often disputed, role of cognition in decision making in team sports (Raab, 2003). The framework proposes that it is important to understand what cognitive mechanisms facilitate decision making in team sport, when, where and why. The work of Changeux and Dehaene (1989), Changeux (2009) have offered a view on the presence/absence of memory representations from the school of neurobiology;

“What is the mechanism that links them together to form a uniquely meaningful entity? This question – known among neurobiologists as the binding problem – is far from being settled” (Changeux, 2009; p. 54).

Similarly, the purpose of this paper is an empirical investigation into what mechanism is used by rugby union players, when, where and why in their decision making. Mahlo (1974) once suggested that a players decision making in team invasion sports, will sit on a continuum between direct perception, a process of analysis and execution, with memory acting as a mediator between perception and analysis. Conversely, Changeux’s (2009) work in neurobiology and neuropsychology has suggested that humans synaptic processes that account for analysis decision making processes, may be too slow to deal with the speed of game situations. Interestingly, this justification forms the conceptual basis for a direct relationship between perception and action offered from the ecological approach to decision making (Raab and Araújo, 2019). Changeux (2009) has championed the presence of memory representations within their work, suggesting that even when situations remove the possibility for conscious thought, representations create neural embodiments of meaning that allow humans to satisfy a situation. That is, even when players rely on intuition and non-conscious decision making when the time to analyse is absent, networks of neurons fire in a synchronized fashion through mental representations that are deemed meaningful over time. This evidence offers the view that meaningful representations, stored in memory are the central mechanism for effective decision making.

In light of the evidence reviewed, one way a deeper consideration of the presence or absence of mental representations can be investigated is to explore different sport-specific contexts within which players are making decisions (Bar-Eli et al., 2011; Birch et al., 2019). The laws of rugby union create game situations that vary drastically in the complexity of information, time available to decide and the desired technical/physical requirements (Tee et al., 2018). Similarly, Raab (2003) suggested that all game moments within team sports possess a level of cognitive complexity, which is defined by the amount of choices, attributes, and time available to a player within a decision making moment. Moreover, he hypothesised that placing emphasis during practice on the development of different forms of capability to act would have specific advantages, which depend on the cognitive complexity of the task environment. Raab found that novice handball players who had developed explicit *knowledge-of* the task were advantaged when cognitive complexity was high, whereas those who had developed *actions-for* rather than *knowledge-of* the task (i.e., implicit learning) were advantaged when cognitive complexity was low (Raab, 2003). Similarly, Basevitch et al. (2019) compared expert and near expert soccer players intuition and option generation in a decision making task. Participants were required to pick a first option and follow up with other appropriate actions to video footage, but were also required respond to occluded images at 400 ms, 200 ms and 0ms in both cued and non-cued environments. Their findings demonstrated that experts intuition and option generation were complimentary of one another across both conditions. At 400ms option generation was deemed more successful, whilst at 0ms and in a non-cued environment, their first option was significantly more successful. Time available seemed to coincide with their decision making process. These findings from Raab (2003) and Basevitch et al. (2019) offer an implication that the application of decision making processes at an individual level may depend on the demands of different game moments (Araújo and Bourbousson, 2016).

Raab (2003) suggested that investigation into implicit or explicit cognitive processes within team sports must consider the role of the environment where one must question whether a cognitive mechanism, whether absent, good or bad, rational or irrational, may be best understood relative to its contextual surroundings (Raab, 2003). Consequently, specific game situations may demand a player’s use of meaningful cognitive mechanisms to make decisions (Raab, 2003; Lex et al., 2015). For instance, the time (Basevitch et al., 2019) and options (Raab, 2003) offered to players by game situations may demand an implicit coupling of perception and action resulting in player’s self-organisation (Passos et al., 2008; Raab and Araújo, 2019), intuition and heuristic driven decision making (Raab and Laborde, 2011; Raab and Gigerenzer, 2015), diagnosis of limited options rapidly (Macquet and Fleurance, 2007; Macquet, 2009) or deliberate evaluation of situational probability and mental rehearsal of possible courses of action (Macquet, 2009; Macquet and Kragba, 2015). Furthermore, at a collective team level, situations of high cognitive complexity may benefit from reference to a *global top-down* shared mental model (Richards et al., 2012, 2017; Richards and Collins, 2020), whilst situations of low cognitive complexity may be best to rest on the *local bottom-up* shared perception of, and response to game information by team members (Correia et al., 2012). Therefore, the objectives of this study were clear; (i) to consider how game situations influence players *perception of information*; (ii) to consider how game situations influence the application of cognitive mechanisms whilst making decisions, more specifically players *actions-for, knowledge-of and knowledge-in the game*; and (iii) to identify the influence of tactics and/or strategy, such as a *common frame of reference*, on player decision making.

Recent research considering player decision making in team invasion sports have adopted self-confrontation interviews to collect meaningful data, which present players with footage of their past actions in order to stimulate verbalisations of their experiences during the event (Macquet and Kragba, 2015; Johnston and Morrison, 2016; Gleeson and Kelly, 2020), such as their lived decision making experience (Varela and Shear, 1999; Macquet, 2009; Bourbousson et al., 2010, 2011). The current study adopted this approach to consider the perspective and thoughts of the player in the context of a single team invasion sport, rugby union (Gleeson and Kelly, 2020; Richards and Collins, 2020). In an attempt to mitigate the fallibility associated with data generated through verbalisation methods (Cappuccio, 2015; Raab and Araújo, 2019), the self-confrontation process was initially framed along a continuum, where players were able to classify their decision making as *No-thought, Fast-thought or Slow-thought* (Macquet, 2009; Macquet and Kragba, 2015; Johnston and Morrison, 2016). Considering the research aims, we hypothesise that individual players would describe their decision making dependent on particular game situations where decisions were an implicit (*no thought*) direct connection between their *actions for* the game and their environment (Cappuccio, 2015; Raab and Araújo, 2019); *fast* and frugal through updating their *knowledge-in* the game (Bennis and Pachur, 2006; Schulz, 2018); or *slow* and deliberate in an offline fashion through their *knowledge-of* the game (Richards et al., 2017; Raab and Araújo, 2019). Whilst from a team perspective, we hypothesise that players would share instances of implicit (*no thought*) bottom-up organisation of players through a shared perception and response to game information (Ribeiro et al., 2019a, b). *fast* and frugal interactions with teammates (Araújo et al., 2015) and *slow* top-down coordination (Steiner et al., 2017) through global tactical principles informed by a shared mental model.

## Materials and Methods

### Participants

Sixteen adult rugby union players from a team in the highest tier of British university (UNI) rugby (*n* = 8) and a professional (PRO) rugby team (*n* = 8) volunteered to take part in the study (see Table 1 for playing positions). The UNI players ranged in age from 20 to 27 years (*M* = 22.5 years, SD = 2.1 years) and had been playing rugby for 7-22 years (*M* = 14.5 years, SD = 4.5 years). The PRO players ranged in age from 19 to 29 years (*M* = 24, SD = 3.42 years) and had been playing rugby for 11 to 17 years (*M* = 14.6 years, SD = 2.3 years). Within the UNI group, two players had previously played professionally whilst another two had represented England students. In alignment with Swann et al. (2015) both groups of players have been identified as semi-elite populations, as the PRO Team participate in the second tier of English Rugby Union, whilst the highest tier of British University rugby union now require’s players to train as full time athletes and regularly results in players progression to the highest tier of Professional rugby. For the purpose of this study findings have been separated by group to identify similarities and differences in accordance with the study aims (Johnston and Morrison, 2016).

**TABLE 1:** Player positions by population.

UNI		PRO	
Position		Position	
Player 1	Full-back	Player 1	Fly half/centre
Player 2	Scrum-half	Player 2	Wing/fullback
Player 3	Hooker	Player 3	Tight head prop
Player 4	Outside centre	Player 4	Hooker
Player 5	Inside centre	Player 5	Hooker
Player 6	Loose head prop	Player 6	Outside centre
Player 7	Wing	Player 7	Second-row
Player 8	Second-row	Player 8	Fullback

### Game Footage

UNI and PRO matches were filmed at the half-way line adjacent to the rugby pitch. The camera was operated throughout to ensure that all players included in the study were identified. Following each match, the individual player’s actions, and the game context (situations) were analysed by each team’s dedicated performance analyst. The player analysis was conducted within 24-h of the game using SportsCode Elite software (V11, Hudl, Lincoln, Nebraska, United States of America). This approach was taken to minimise the disruption of player’s typical interaction with performance analysis between games and because in-house analysts are better able to perceive individual and collective decisions and actions in reference to their teams’ tactical approach, whilst utilising repeatable methods of analysis. For every decision identified, the analyst clipped the footage 5 s prior and 7 s after the decision in order to capture the situation leading into and the outcome of the action, respectively. On occasions when actions lasted longer than 7 s the clip was extended. Video clips for each player were compiled into a film in the order they occurred during the match. For the purpose of data collection, each player’s video clips were then edited into a changed order using Adobe Premier Pro video editing software (Adobe, San Jose, California, United States of America) by the lead author. The rationale for this was to monitor the consistency of player’s decision classifications (Kinrade et al., 2015) and to reduce a possible learning effect between stages of the procedure.

### Procedure

The data collection procedure took place in a mutually agreed, quiet location within 48-h of the final whistle of each game. UNI and PRO players completed this process twice (*n* = 32 games). Within the procedure, players were required to complete three stages of data collection; (i) unstimulated recall of involvements, actions and decisions made within the game; (ii) decision classification; and (iii) a repeat of decision classification alongside a self-confrontation interview. Each stage of the procedure was recorded using a dictaphone.

#### Stage 1: Unstimulated Recall

Prior to watching any game footage (Gleeson and Kelly, 2020), players were first asked to verbalise their involvements, actions and decisions within the game. The rationale for collecting unstimulated data was to identify what actions and decisions players could recount from the game without prior stimulation from game footage. This method served a clear purpose, as it enabled a consideration of the trustworthiness of the data captured during stage 3 of the procedure (Bourbousson et al., 2011). Furthermore, unstimulated verbalisations could then be cross referenced to stimulated verbalisations of the decision process during the same game moment.

#### Stage 2: Decision Classification

On both data collection occasions’ classifications of No-thought, Fast-thought and Slow-thought were defined for the players by the first author. No-thought classifications were defined as *a decision where no conscious thought took place in leading to an action*, Fast-thought classifications were defined as *a rapid conscious decision leading to an action*, whilst Slow-thought classifications were defined as *a slow deliberate conscious decision where a number of options are considered leading to an action*. Following this, players had the opportunity to ask clarification questions before beginning the video classification task. The task commenced when players were shown a video of their actions and decisions in the order they occurred during the game. Each clip was played once, and the video paused at its conclusion where the final frame remained on the screen. This initiated players to classify using a second laptop (with screen occluded) whether the clip represented a *No-thought*, *Fast-thought* or *Slow-thought* decision. Players had a 5 s limit to classify each decision before the researcher played the next clip (Mulligan et al., 2012).

#### Stage 3: Self-Confrontation Interviews

After responding to all clips once, the players repeated the decision classification process by watching the same clips presented in a different order. In the second round of decision classification the 5 s time limit was removed. Instead players were asked to verbalise how they came to their decision through self-confrontation elicitation interviews (Von Cranach and Harré, 1982; Feigean et al., 2018; Gleeson and Kelly, 2020). Within self-confrontation interviews players are likely to digress to reflect-on action or self-rationalise and justify their decision making (Gleeson and Kelly, 2020). Therefore, the interviewer encouraged the re-enactment of their lived experiences during the game via prompts such as “why slow/fast/no thought?;” “what are you thinking?;” “what information are you responding to?;” or “talk me through what happened?”.

### Data Analysis

The reliability of the UNI and PRO players No-, Fast- and Slow-thought decision classifications between stages 2 and 3, were confirmed by calculating the intra-class correlation coefficient (Chronbach’s alpha: UNI = 0.92; PRO = 0.89). For the qualitative data, all interviews were audio recorded and transcribed verbatim. Each transcript was read numerous times to ensure familiarity and understanding (Taylor and Collins, 2019). Due to the large amount of qualitative data drawn from the unstimulated and self-confrontation interviews, the data was analysed in two stages. The first stage employed qualitative coding and theme development procedures commonly used in sport coaching research (Braun et al., 2016; Miles et al., 2020; North et al., 2020). Initially, verbalisations were attributed to Unstimulated responses and No-thought, Fast-thought and Slow-thought decision classifications. Within each component, the unified conceptual framework for decision making in team invasion sports was employed to conduct a deductive content analysis (Johnston and Morrison, 2016; Kyngäs et al., 2020). This allowed a hierarchy of deductive themes to emerge - *perception of information*, *actions-for*, *knowledge-of, knowledge-in* and a *common frame of reference –* which helped comparisons within and across populations to be made. Analysis was enhanced by using qualitative software (QSR NVivo12). To examine the relation between the frequency of No-, Fast-, and Slow-thought decision classifications made within each deductive theme by each player group chi-square tests of independence were performed.

The second stage explored each prescribed theme deductively to abduct “elementary units of meaning” in relation to each study aim (Thereau, 2003; Macquet and Kragba, 2015; Gleeson and Kelly, 2020). Furthermore, units explored player’s experiences of cognitive, perceptual, psychological, sensorimotor, physical and environmental events, inclusive of the game situation, teammates, opposition and the coach (Varela and Shear, 1999; Gleeson and Kelly, 2020). Where possible, codes were created in line with cognitive mechanisms underpinning the unified conceptual framework for decision making in team invasion sports (Ashford et al., 2021). Thus, due to the range of verbalisation data collected, inductive reasoning was used to abduct data that could not be satisfied by underpinning deductive themes. Again, results were compared across populations (Johnston and Morrison, 2016).

In accordance with recommendations by Tracy (2010), the *credibility* of the data was appraised through triangulation of quantitative decision-classification and qualitative self-confrontation interview data (Macquet, 2009). To safeguard *sincerity* in our approach a critical friend, independent of the authorship, was invited to review, comment and edit our deductive analysis and construction of elementary units of meaning for both UNI and PRO groups of players (Smith and Caddick, 2012; Cronin and Lowes, 2016). Finally, *rigour* was elevated by only including players who completed the full procedure twice^1^ and by conducting interviews within 48 h of the final whistle of the game in an agreed and consistent location (Tracy, 2010).

## Results

**FIGURE 2:**
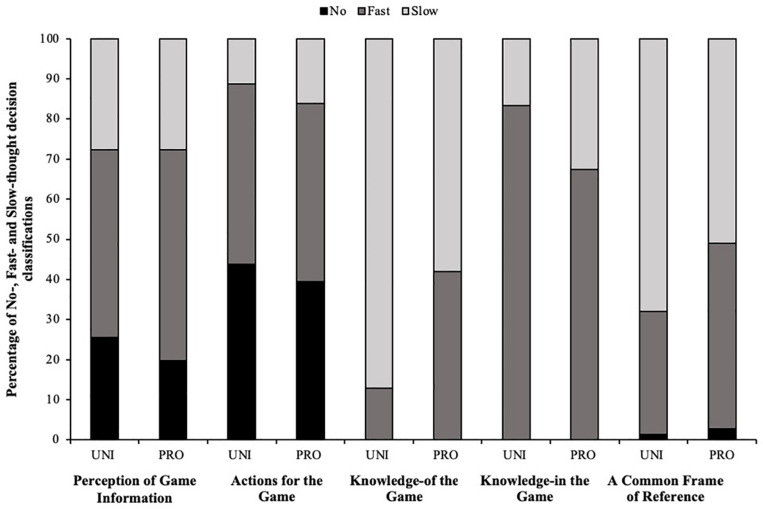
Percentage of No-, Fast- and Slow-thought decision classifications for UNI and PRO groups for each deductive theme.

To signpost the reader through the results, numerous examples of raw data have been included in Tables 2-4. Firstly, Table 2 displays elementary units of meaning constructed from Unstimulated verbalisation data, where player’s recollections were aligned to their decision classification and stimulated verbalisation during stage 3 of the procedure. Figure 2 presents quantitative findings, comprised of a deductive content analysis, specifically the collation of player’s decision classifications and references to each deductive theme; the perception of information; actions-for the game; knowledge-of the game; knowledge-in the game and a common frame of reference (Ashford et al., 2021). Table 3 demonstrates elementary units of meaning constructed to explore the decision making process and cognitive mechanisms described during stimulated verbalisations of decision making processes. Finally, data emerged, displaying how four distinct game moments in rugby union; the lineout; the scrum; defensive situations and offensive situations^2^, are commonly associated with distinct forms of decision making processes (see Table 4) as hypothesised by Raab (2003). However, the association is not just complexity, but also time available.

**TABLE 2:** Thematic analysis of Unstimulated verbalisations, stimulated verbalisations and decision classifications of the same game moment [*number in brackets denotes the number of player references to this theme*].

Unstimulated Raw Data example	Stimulated Raw Data Example	Decision-Classification	Deductive Theme	Elementary Unit of Meaning
“We said look at front door options instead of going out the back. Cause they were sort of chopping off us and biting on us, so thought we’d put it into an open play move I hit **** on a short ball and he went through and put **** in and scored.” (UNI Player 4, Game 2)	“I was initially looking outside me, as I thought that’s where the support would be coming from as we had pre-called that it would be a hit. It was a pre-call pat hit. **** should be an option, but once I make the break I look outside straight away.” (UNI Player 4, Game 2)	Slow-thought	Perception of information (50)	Defensive picture
“Next play we’re all spaced up and I fly upon to the last man and then he gave a pass away and I had to shift off.” (PRO Player 2, Game 1)	“I always assess the first guy on the lead and then push off onto that shoulder ball I was a little slow on that and then it was the case of my winger overselling himself, and in recognising that I knew I had to chase back.” (PRO Player 2, Game 1)	Slow-thought		Offensive picture
“I could see there was a bit of space on the outside which I saw the winger had chased in on me. Went to look to go either side but he’d actually closed me off really well so I fended him off to get me a bit of space and span into where the forward were all stood and opened up.” (PRO Player 2, Game 1)	“I didn’t think I would beat him for pace because he was backpedalling and definitely had the angle on me. They had cover, I knew I could beat him one on one but was very much aware of the cover chasing me, so attempting that would slow me a bit, so my thought process was let’s keep us on the front foot.” (PRO Player 2, Game 1)	Fast-thought		Opponents movements
“There was one missed tackle where their winger broke through and me and James both sort of went for him at the same time and both missed it.” (UNI Player 1, Game 1)	“He broke the line and **** sort of was in behind and he was shouting at me I’ve got it I’ve got it so I sort of stepped out the way and then as he missed him went for him, so my process was quite slow” (UNI Player 1, Game 1)	Slow-thought		Teammates movements
“We could of thrown to the back of the lineout but we haven’t had any success with the back ball.” (UNI Player 8, Game 1)	“The **** ball hadn’t been on all day which is the back ball on the lineout which we really haven’t been hitting to much anyways & it’s wet and rainy so it’s a much higher risk” (UNI Player 8, Game 1)	Slow-thought	Actions-for (119)	Self-awareness
“So whenever I noticed him being close to the ball I just ensured I was there quickly to blast him out, or make sure that when he was tackling me, when I was on the floor to constantly make a second effort so he couldn’t get hands to the ball.” (PRO Player 5, Game 1)	“In the build up to it, just before we get the ball (pod of three), I’m looking at the defensive picture for the red scrum cap. The risk goes up when he’s there.” (PRO Player 5, Game 1)	Fast-thought		Intentions
“We had a scrum just before half time, which didn’t go as well – one of the worst scrums of the game – we didn’t get out the blocks very well.” (PRO Player 3, Game 1)	“So the hit doesn’t go to well, and then he’s sort of on the outside, and I’m thinking at this time about getting forward so he can’t get around me.” (PRO Player 3, Game 1)	Slow-thought		Outcomes
“Before the game I’d noticed that **** their 7 was really strong over the ball, so whenever I noticed him being close to the ball I just ensured I was there quickly to blast him out, or make sure that when he was tackling me, when I was on the floor to constantly make a second effort so he couldn’t get hands to the ball.” (PRO Player 5, Game 1)	“It just became a thought process of if he’s there just smack him and get him away. If I went over the top of him there, he’s in a strong position and he just wouldn’t of moved. It was definitely a conscious decision to get my head under his shoulder and just smack him off the ball. My thought process was jacked by his red scrum cap.” (PRO Player 5, Game 1)	Fast-thought	Knowledge-of (19)	Opponents
“And then defensively in the lineouts we stole, from me jumping at the front and then there was one other that **** got his finger tips on then obviously I remember the last 6 minutes in pretty good detail.” (UNI Player 8, Game 2)	“So we decided before the game to line up slightly in front of their jumpers. Then the picture I see makes sense as they’re just going quick and to the middle I think it was their front jumper who turned deliberately” (UNI Player 8, Game 2)”	Fast-thought		Roles & responsibilities
“First thing was the kick chase of the restart so we knew previously that they were going to go long so set off, kept the line and I could chase out.” (PRO Player 2, Game 1)	“I’m going up I’m looking at who’s around and who’s catching it because they’re going to do different things, because if it’s a 9 catching it I know has going to do something different to a bigger guy.” (PRO Player 2, Game 1)	Slow-thought		Tactics
“There was one instance where the ball came to me, but the defender was on me so I had to tip it on really quickly.” (PRO Player 3, Game 2)	“As I got the ball I saw that **** hips instantly turned into toward me. So I gave the ball to **** to gain a better outcome. (PRO Player 3, Game 2)	Fast-thought	Knowledge-in (5)	Change in expectations
“My decisions following that point was to think ‘right, let’s keep it simple’ and as a lineout caller I tended to back myself a lot. I was like right ‘I know my shit’ so I’m going to call it to myself.” (PRO Player 7, Game 1)	In this game we had to get to trusting what we were doing, the fact that it was personnel messing up, i.e., a not straight, gives you confidence – it’s not like your calling is wrong.” (PRO Player 7, Game 1)			Game context
“It went well, yeah we Ginned off me. Then there was a lot in midfield in this game and we went into the game trying to win the midfield.” (UNI Player 2, Game 1).	“I’m not reacting to anything, I know we’re in midfield on the half way and really that’s a game plan type thing it’s like we’re on the half way they’ve got a good defensive line so.” (UNI Player 2, Game 1).	Slow-thought	Common frame of reference (28)	Tactical rules
“I stayed hugging the touch line and called flash which is our call to jump back down on the blind side.” (PRO Player 2, Game 1).	“Saw it was on, called the flash my options were to flatten up or stay deep to get the ball and make that decision as easy as possible for our ten, basically I could see that their was a 6 in front of me, and I knew I could beat him into that space.” (PRO Player 2, Game 1)	Fast-thought		Common language

The **** denotes names and terms that have been removed for confidentiality and anonymity.

**TABLE 3:** Thematic analysis of UNI and PRO Rugby Union players verbalisations of their decision making process [*number in brackets denotes the number of references made to theme*].

Population	Classification	Deductive themes	Meaningful Units	Raw data examples
UNI	No thought	Perception of information (45)	No time (5)	“That’s no thought again, sort of turned at the last second didn’t have time to think.”
			Opponents movements (22)	“Because it’s just like, he changed direction so you’ve just got to make it.”
			Teammates movements (7)	“**** made contact with him and I just assume he would and then he got passed and I’ve just got to hit him. It was an impulse decision.”
			The ball (10)	“Just the balls there, it’s bouncing around like a pin ball so just dove on it. I can’t even remember doing that after the game.”
PRO	No thought	Perception of information	Audio (3)	“Just heard something and reacted with an offload.”
		(56)	No time (10)	“I didn’t have time to look up to scan, which put us in an awkward position.”
			Opponents body position (6)	“There’s no real thought there, was just get over it, deal with any threats and get low.”
			Opponents movements (19)	“That’s just a reaction, he’s come down the channel, can’t really explain it more than that.”
			Teammates movements (12)	“We’ve got a big emphasis on ‘pushing through’ at the moment, so when I see a break like this one it’s almost just instinctive to sprint and follow.”
			The ball (6)	“There just no logical decision here, I’ve not assessed the actual ruck area, I’ve just seen the ball and gone for it.”
UNI	Fast thought	Perception of information (110)	Audio (4)	“The thought behind it is the 9 has picked to go left and then I’ve heard the bounce back call, so that’s given me time to get up.”
			Opponents body position (3)	“I definitely thought about how I was going to clean him out. But that was at the last second when I hit him, I hit him and he didn’t move so I had to react and roll him out.”
			Opponents movements (39)	“So here I’m thinking he passes to me and I go under, but I didn’t see the 14, until the last second which is when I gave it. I only really had two options to pass or have a go, but it changed from have a go to pass at the last second when I saw the 14.”
			Teammates movements (24)	“Bit of a fast thought. Initially it wasn’t my tackle, so it was whether to go in or not. I see that it’s a semi dominant carry so I had to go in to help out.”
			Defensive picture (24)	“Space was there, no need to wait and give them time – let’s just go.”
			Offensive picture (4)	“I think I seen, I looked behind the line so I knew that they had no real options on outside, so I shot up a little bit, it was just fast thought to shoot up a little bit.”
			Time (12)	“It is my job to look out for the overthrows and tips but not always expecting them to stuff up their line outs. When it does though I’ve got like a split second to react. My first response is always to just carry.”
PRO	Fast thought	Perception of information (164)	Audio (6)	“Also I heard as I took a step forward the ball player out the back say shadow as he’d obviously seen a clear opportunity to act.”
			Haptic (3)	“So the hit doesn’t go to well, and then he’s sort of on the outside, and I’m thinking at this time about getting forward so he can’t get around me.”
			Opponents body position (20)	“Just remember looking at his body shape, there just wasn’t an easy place for me to put a shoulder in, it looked like I might simply hit and bounce off. Which led me to think, right let’s just wrap him up it’s the safer option. I’ve only got the ability to go through this thought process because of the time that’s available when I’m chasing. But I’ve put this down as fast thought as at the last second I knew I couldn’t get to the ball. As the kick was coming down was where I had to make the decision of when and how to tackle.”
			Opponents movements (25)	“Again that’s a similar sort of thing, my first decision is to join or stay away depending on which side he steps, or where he runs, indicated whether I go in or not.”
			Teammates movements (24)	“I remember wanting to pass to the fullback coming short but he stayed wide, which meant my decision was made for me, so I pumped and bought the ball back in and tried to run into the gap myself.”
			Defensive picture (26)	“But as it developed I can remember their tail man at the lineout looking directly at our 9. Which if he chases leaves a nice healthy gap for me.”
			Offensive picture (20)	“It’s a fast thought process because I’ve seen that they had a lot more numbers than we did on that side of the ruck so I’ve just arse ended across and continued to go across.”
UNI	Slow thought	Perception of information (95)	Defensive picture (26)	“Cause this one was more there wingers were dropping really deep as they thought we were exiting. I was looking up every time I received the ball to see how deep their wingers were on each touchline. They had a minimum of two players drop back each time and just kick it long and push both wingers backward instead of having them both run onto the ball.”
			Offensive picture (13)	“Their numbers up, the defensive set up we’re going to have to wedge. They’ve got 21 coming short, so I stick on him and then the ball goes so I wedge off”
			Opponents movements (18)	“It was my man, sort of lined him up but just yeah he’s always my man, I’ve just shot up a bit and yeah it’s a slow thought. Because I’ve had 5 seconds to hit him, sort of when you’re in the line waiting for the ball to come out I was thinking I’m going to hit him, he’s my man sort of thing.”
			Teammates movements (13)	“That was just us running a Nike – **** was running a hard line and then one of their lads jumped out and **** was the only option in my head.”
			Time (25)	“We had our line set and I saw him calling for the ball, so I had time to get my head up and make the tackle. Yeah, I knew that that was my tackle.”
PRO	Slow thought	Perception of information (99)	Audio (11)	“So I hear from the outside that **** calls ‘chilli’ so I look up and see we’ve got numbers. So as I get the ball I’m looking to see how the man I’m engaging reacts, but he stay’s on us so I make the pass. If he hadn’t of stayed on us, I would have carried into the space. ‘Chilli’ pretty much means lets move the ball wide because there’s space.”
			Defensive picture (16)	“Another slow thought, as I’ve pre-called the lineout. They gave me the space where I called it so I took it.”
			Offensive picture (17)	“Erm, I’ll say slow thought for that one. 2. I was quite unsure of who my man was. I didn’t hear too much communication from inside me so I had to stay a little bit longer than I would have liked before I could push off and get to the shadow. He was quick as well which made it tougher. Yeah so, slow thought didn’t have much from inside me, didn’t have much from outside me so I had to process all the information myself to make the decision.”
			Haptic (8)	“Trying to maintain shape and working on my shoulder are the things I‘m consciously working on and in my set up working with ****, he’s a really good prop if he moves forward, but If he moves forward he struggles, so there’s a lot of talk with him and a lot of set up with him before trying to get him forward, so I can get him through, we’re always saying get your inside shoulder through and your left foot up so he’s nice and square so he can get through.”
			Opponents movements (14)	“I’m assessing whether the 9’s running, no, get out and get on his inside, and then I just folded round the corner when asked.”
			Teammates movements (15)	“But then it changed as the pass went to him instead and it disrupted things. Really, he should just carry, but instead he goes ahead with the plan and tips the ball on to me and I don’t really want it, so I drop the ball. I mean its poor skill from me I should still take it. Because I expected something different, its resulted in a mistake.”
			Time (18)	“That whole time I was assessing the options that were available. Where we need to press, who’s inside me, I think we were out of backs as I only had forward inside me so this changed the way I defended, as I was a lot more passive to give these guys time
UNI	No thought	Actions-for the game (37)	Reaction (33)	“Erm, because I didn’t see him, so I just turned and he was there so I just hit him. He had the ball so, that was my instinctive response in defence.”
			Rehearsal (4)	“It’s like if I’m one on one 90% of the time I’ll go off my left foot because 90% of the time I’m looking like I’m going to go in the other direction, so the defender just turns. Is that cause you’re better off your left foot. Yes, definitely I know that. I just spend all my time getting people to go that way so I’ll just go the other way.”
PRO	No thought	Actions-for the game (57)	Reaction (52)	“No thought, he fumbled it, then it just kind of happened. I had no idea what he was going to do because he’d fumbled it, so I just shifted it away straight away.”
			Rehearsal (5)	“That’s just me following my process – we’ve rehearsed where he should be so I just throw it.”
UNI	Fast thought	Actions-for the game (55)	Self-awareness (12)	“Well there was either two options there for me, carry or pass. I seen that winger back so I gave it, it wasn’t pre-planned or anything.”
			Intentions (41)	“You see that they’re setting up for the pick and go, then you’re just waiting for them to move. You know what you have to do and you’re just responding to their body position. Just waiting for them, which triggers the reaction.”
			Misjudgements (2)	“He did a really good job and he didn’t bite in, so I tried to take the outside centre on the outside and as I seen him bite in I tried to release the ball out the back and the ball got ripped off me.”
PRO	Fast thought	Actions-for the game (83)	Self-awareness (27)	“I know I’m not the best jackler and I know I’m not the best at counter rucking, so the more time spent driving the player back the more opportunity to slow the ball down.”
			Intentions (46)	“When I do hit the ground I’m thinking ‘second movement’ as I have to ensure I get the ball back cleanly, this is something that I just have to do.”
			Misjudgements (10)	“We need a few more men, the ball came out messey, and whilst the ball was in the air I decided to go for it rather than cover on the outside. Went in for the ball tried to be smart and absolutely pinged it up and forward.”
UNI	Slow thought	Actions-for the game (26)	Self-awareness (9)	“Again I think I had quite a bit of time to think about it. Because they were passing wide I just had to keep going off them. I always back myself, like show them the outside and execute the tackle basically.”
			Awareness of teammates capabilities (2)	“Even though their winger was relatively quick he broke the line and **** sort of was in behind and he was shouting at me I’ve got it I’ve got it so I sort of stepped out the way and then as he missed him went for him, so my process was quite slow as I could hear **** saying he had the player.”
			Intentions (15)	“It was more or less the first couple of minutes of the game so for me I’m thinking I need to get that tackle I need to get that first hit in so if I do that I can get into the game pretty well.”
PRO	Slow thought	Actions-for the game (40)	Self-awareness (20)	“I know I’m getting the ball. Had time to think about the pump of the ball, create myself some space and then drive.”
			Intentions (20)	“I’m constantly feeling what our hips and shoulder connections are like, when I call the seconds in where they are so they’re in good positions most of the time it’s fine, if they’re not I’ll ask the referee to blow it up and reset.”
UNI	Fast thought	Knowledge-of the game (22)	Opponent (3)	“From watching their game film before this game, I knew that they liked to go quickly on their five man set up. So we decided before the game to line up slightly in front of their jumpers.”
			Roles & Responsibilities (7)	“In terms of when we’re coming round the corner of the ruck, we set up in guard, 9, 1, on defence I’m just then waiting for them to come into my channel. I should have come up there but I’m just waiting to see if that’s switch happens. Once I see that they’re getting the ball then my brain jogs and I’m reacting.”
			Self-awareness (1)	“I go through the same process at every ruck, first thing I do is find out what’s happening, find out where the ten is, where the forward are, first thing that goes into my head is what are we doing? The two three forward there hitting hard and then who’s going to be out the back. Some people like looking at the gaps first but I also think, by the time you get there they’re going to have closed that. As my hands go on the ball then, I have a look at the gap available and then I go for it or the pass.”
			Tactics (11)	“Once we get to the edge, we tank back in. We do it all the time in training so it’s just a rehearsed rule we play too.”
PRO	Fast thought	Knowledge-of the game (49)	Knowledge of opponents (5)	“So, ****, their hooker, I’ve played him before – he doesn’t like scrummaging under your chest. He obviously enjoys going into the gap, so my thought process was to make it uncomfortable for him. Turn my right shoulder down and put pressure onto his neck.”
			Self-awareness (3)	“I definitely know my strengths, I know when I should and shouldn’t go for a jackal, when I’ve actually assessed the situation. If he falls on his back or exposes his body, that gives me a better opportunity to go for the ball.”
			Knowledge of teammates (2)	“He’s a very good offloader, so I knew that his first instinct would be to give the ball so I jumped at the chance haha.”
			Roles & Responsibilities (19)	“Here I saw they had got quite far along at the point of contact, so I had to call two round the corner to defend. I think this call is because we do a lot of it in training and we’ve had numerous bollocking’s over it as well about being early. I think like we made it so clear, my coach said you might be calling them but they can’t see it, so be really dramatic about it, wave your arms, pull them and I knew as well that because they were so far along that we’d definitely need two because their attack had cut off most of the pack. We have a clear policy of make the space for them to fill it.”
			Tactics (20)	“I was in position and I know our strategy there is to hit the middle man, he’ll either carry and give a tip to me and in this instance I’ll just clear that ruck there. Unless we’ve hit a guy that we usually wouldn’t hit or they tip it on. Normally, so if there’s 3 this guy clears the outside and this guy clears the inside so if it changes that’s when we have to make the decision to adapt.”
UNI	Slow thought	Knowledge-of the game (83)	Knowledge of opponents (4)	“Because from the preview of the game I knew where they tend to give up space defensively in the lineout so I knew where we wanted to attack.”
			Roles & Responsibilities (30)	“Just because of time, defensive structure, wedge, on the inside of defence, kind of know where they’re going and the tackle I need to make.”
			Self-awareness (10)	“I heard the tank call, so I had time to look up and see what I’d run. I was always going to run there, I hardly ever do look for options, I just go unless there’s a call for a tip, but yeah when I do get the ball I’m pretty tunnel vision like.”
			Tactics (34)	“Game plan related one, we’re close to our own line – the decision was to exit so got some blockers in and I knew I was hitting **** so, despite it being at a bad angle.”
			Teammates (5)	Well I can see he’s got the ball and I’m kind of deciding when to make the tackle or him to make the tackle I’m talking to **** on the outside telling him to wedge so I’m making the decision as it unfolds. It gives me more time to weigh up the situation and make a tackle rather than if I fly into that there’s a risk of it coming off bad.
PRO	Slow thought	Knowledge-of the game (88)	Knowledge of opponent (9)	“We identified him as a threat as he got three turnovers in the previous fixture so. It wasn’t something we’d prepped as a team, it was something I’d noticed from watching the video, like everyone will know we’ve got to watch him cause he’s going to cause us some trouble if we don’t look after him. It just became a thought process of if he’s there just smack him and get him away.”
			Roles & responsibilities (38)	“At 99% of mauls my role is to get the ball at the back, so there’s no thought process its just my job. Then it just becomes slow thought based on my feel of how the mauls progressing, is my binding tight, are we low enough, is the ball on the right hip, it becomes a check and challenge because you’ve got the time to do it.”
			Self-awareness (5)	“It’s just the time I’ve got to set up, I’m at the back here and we’re further up the pitch so I’m not expecting it at the front. Then it’s a case of connecting with the line, I got caught on this last week as I was too far ahead, it was definitely a mistake I was aware of this week and It was definitely conscious as I made my decision.”
			Tactics (30)	“Slow thought, same premise really it’s a set move, we both know that we’re running hard at the line, all I’m reacting to is catching the ball, but I know that I’m running that line and picking that hole on that side of the defender because I have time to. The catch is more the skill than the decision.”
			Teammates (6)	“Knowing that I’ve got **** and **** chasing with me allows me to go at their pace and knowing all the time that we can put pressure on him and contain what we can.”
UNI	Fast thought	Knowledge-in the game (85)	Change in expectations (57)	“Again similar as before, it was the other man’s tackle but he’s stepped into my way, so just reacted to that.”
			Game context (14)	“Erm, I think I’ve made a bit of a mistake there as I’ve got passed him and he’s just stepped in and I’ve just reacted to almost a panic tackle to get him down. That was probably happening a bit quicker and like a said probably a bit of panic. The context of the game as well as they were coming back into it as well.”
			Risk/Threat (14)	“Just so he couldn’t get up and go again, cause if I don’t go in there he’s just going to get up and keep going, but if I put a knee on him I kind of I just stop him and get back out.”
PRO	Fast thought	Knowledge-in the game (86)	Changes in expectations (46)	“As we’re coming round the corner here, I’m not expecting him to be tackled, so I quickly thought ‘right I’m going to hit this as hard as I can’.”
			Game context (15)	“I think we turned it over so, for them they were set and ready to attack so all I knew was I had to get into the midfield and get it out to the wing and we’ll probably get some decent metres on the edge.”
			Risk/Threat (25)	“But then because my teammates get’s hit and turned there’s a threat of the ball being turned over. So, then I became conscious that I had to identify the threat and deal with the threat quickly.”
UNI	Slow thought	Knowledge-in the game (18)	Change in expectations (9)	“Well I had time to think, so it was like I caught the ball quite didn’t catch the ball very well as it was kind of behind me and then it was just kind of flat D on us.”
			Game context (3)	“I simply saw a man that I thought I could beat. It was raining, the ball was really greasy and wanted to hold onto it.”
			Risk/Threat (6)	“He was a little bit of a threat, it was his first carry so and my first tackle, first involvement didn’t want to fly out the line. Didn’t want to try and put in a big shot and end up on my arse if you know what I mean. He only had one or two outside, there was definitely one outside.”
PRO	Slow thought	Knowledge-in the game (42)	Change in expectations (10)	“I suddenly realised that I was too far ahead of the defensive line and realistically I should be a metre of so deeper so I’m more connected in the line. Once that pass happens, I know I need to get back and make that tackle.”
			Game context (22)	“Again this was a case of awareness on the field. I’m not wanting to take the ball to get personal glory out of what is a no win situation so told him to carry again, and knew I had to hit that ruck as it’s the wide ruck and the wingers always in it.”
			Risk/Threat (10)	“So I knew from looking in front of me that that’s a back, so I’m talking to the man outside of me telling him to stay with us, because if he has a crack at me he’ll probably do me for pace.”
UNI	No thought	Common frame of reference (1)	Common language (1)	“Erm the called a hippo shadow but he came up very quickly so I had to sort of, I didn’t even see him until the last second and then I just flicked it on. Just saw and acted really.”
PRO	No thought	Common frame of reference (6)	Common language (2)	“That’s just simply getting the ball away from the point of contact, in other words ‘get a shift on’. Not thinking really, we have what we call a ‘two pass policy’ on turnover to try and get the ball away from where they’re heavily populating the pitch.”
			Tactical rules (4)	“I hit the ruck on autopilot”
UNI	Fast thought	Common frame of reference (46)	Tactical rules (27)	“That’s a fast thought, if 9 doesn’t go in we tank, if 9 does go in we power, but we have said that that is something we could do differently. So if 9 goes in we could pass off the base, just a little short one to get outside the tight guys. That’s what we prepare for in training all the time, so there isn’t much thought that goes into it.”
			Common language (15)	“It was a pre-call pat hit. Because **** should be an option, but once I make the break I look outside straight away, so that’s my initial thought on the pass.”
			General communication (4)	“**** told me just to carry so I just carried, it spilled out the scrum so I just thought set a phase we can go again.”
PRO	Fast thought	Common frame of reference (89)	Tactical rule (50)	“We have a tackler + 1 rule, always two in the tackle, because he did step on the inside, my next thought was just to whack him and try and knock him back.”
			Common language (29)	“At the time, **** had called two, with his fingers in the air, which meant they wanted two players to fold round the corner so I knew I had to get to ‘20’ and I was telling my man on my inside that he was at ten. Then they know that my next job and role is to watch the 9”
			General communication (10)	“I called for the offload here of **** rightly or wrongly”
UNI	Slow thought	Common frame of reference (92)	Common language (38)	“Slow thought on that one, my positioning is dictating where he’s going to run, which gives me time to select which tackle I’m going to make and it happened in that way. It wasn’t like I’d just got up to make a tackle. We were in a wedge, didn’t mind about the space we conceded just making the tackle. He was a little bit of a threat, it was his first carry so and my first tackle, first involvement didn’t want to fly out the line. Didn’t want to try and put in a big shot and end up on my arse if you know what I mean. He only had one or two outside, there was definitely one outside.”
			General communication (6)	“When I caught the ball again, if I remember there were a lot of players calling outside me shouting that I had time on the ball, perhaps I could have given that wide instead of trucking it up the middle myself.”
			Tactical rules (48)	“I’m not reacting to anything, I know we’re in midfield on the half way and really that’s a game plan type thing it’s like we’re on the half way they’ve got a good defensive line so, what’s the point in attacking them from here when we haven’t really got any momentum, had a blocker in so it was like **** was ready for it, tank was set up so just went through the process for my box kick.”
PRO	Slow thought	Common frame of reference (111)	Common language (44)	“Here, ‘pepsi’ was called, which was our call for a box kick, which meant we migrated to width and plugged any gaps so our kick chase was effective as possible. Just becomes habitual.”
			General communication (9)	“I’m talking to him again, telling him to carry I know that if I carry it I could be pushed into touch.”
			Tactical rules (58)	“The ball in that situation is pre-programmed to go to him and we had spoken about me taking a tip on to give us more width before that phase unfolded as we noticed they were quite spaced apart in their defensive line.”

The **** denotes names and terms that have been removed for confidentiality and anonymity.

**TABLE 4:** UNI and PRO Players verbalisations of decision making processes in response to specific game situations.

Game Situation	Decision Classification	Meaningful Unit(s)	Raw Data Examples
Lineout	Slow-thought & Fast-thought	Common language Tactics & Tactical rules Defensive pictures Time	“Queen was my only real option, because my original call was a front ball. On the six-man spread, Jack is slow at the front cause we have to step to and they have a pod set ready. I think a lot of these judgements are just drawn from previous games, having tried this before. Like I could go check out Jack on a six man, even if they’re set and we’re taller they don’t have to move anywhere, they can just go straight up if they’re patient.” (*UNI Player 8, Game 2*). “That was more of a fast thought. Because we were going to go with a lineout call, but **** see’s that one of the check outs was on so, every lineout we go in we’ll go in with what we call a ‘movement call’ and then if **** call’s check we all go to that movement.” (*PRO Player 3, Game 1*) “Because from the preview of the game I knew where they tend to give up space defensively in the lineout so I knew where we wanted to attack.” (*UNI Player 8, Game 1*). “This one is slow thought because it’s a lineout call. I’ve got time to think through the process and think about how they’re setting up and which of our calls are likely to work best. Lineout calling is like a game within a game, in that I’ll look at their game video in the week leading up to the game and see how they defend. Then I know what options I want to use, off the back of that each line out then goes onto the next. So we’ve used this lineout and its worked well, then I know that they’re following the dummy pod back – so that will set me up for another one. So you use one to set up another one and so on and so forth. I’d liken it to a game of chess almost. (*PRO Player 7, Game 1*) “But then I saw that they’d set up 3-1-3 defensively almost, so that they have the people there to sack it right away but in the middle on our queen ball they only had one person and they’d already disrupted our maul throughout the game already so I decided to go for the queen ball because they only had one person to stop us, meaning it was more likely for us to get some go forward.” (*UNI Player 8, Game 2*). “Pre-called it, we walked in and from the way they’d set up, there was no check outs on. When I walk into the lineout with my hand up it’s a signal to the hooker that I’m going to call now. Our trigger for the movement is me to call ‘check’. They’d know that If I said it, then they would know that the ball wasn’t coming to me. When I put my hand up, then the hooker knows, or people know that we’re checking out.” (*PRO Player 7, Game 2*). “So I had time to assess the options, there was no rush and no need for tempo. That was basically the game plan, we knew what we wanted to do and where we wanted to attack going in and the option was there on that occasion so.” (*UNI Player 8, Game 1).* “I’ve got time to think about it. As I said on the last one, the reason why, I’ve bumped him back and gone in behind them. It’s a case of working out what they’re doing and almost predicting what we’ve seen of them on video from recent weeks. In this game we had to get to trusting what we were doing, the fact that it was personnel messing up, i.e., a not straight, gives you confidence – it’s not like your calling is wrong, like where you’re expecting it to be on and then its not.” (*PRO Player 7, Game 1*)
Scrum	Slow thought	Haptic Tactics & Tactical rules Common language	“On this one we were quite well set, but we don’t win the hit, so we’re in good shape and we’ve got good pressure but because we don’t get through it was a bit of a struggle. So their lose head steps back and their tight head steps up and then we had that whip in the scrum.” (*PRO Player 3, Game 2)* “I just go through my processes, based on how it feels, If I feel dominant I’ll look to be aggressive, if not I’m a bit more reactive.” (UNI Player 6, Game 1) “It almost goes a bit like a checklist, so when you go down on crouch you go for your bind, I’m always looking to win that, that way you’ve got him sitting on his heals. Then I’m looking to crash through the marks, win the race across the gap and get that 1, 2. Lock the scrum down and put him in bad shape. We spend a long time prepping them through video footage, so **** will step out and he’s got a negative bind so my intentions were to clamp him down so he couldn’t get his arm up, so he was already in a bad position. (*PRO Player 5, Game 1*) “It’s a slow thought here, we get the free kick at the scrum and **** asks us whether we fancy a scrum, so we go for a pen. Our call is ‘Argie’ which is all out attack at the scrum and we get a penalty.” (*PRO Player 5, Game 2*).
Defensive situations	Slow thought	Offensive pictures, Tactical rules, Roles & responsibilities, Common language.	“I hesitated here, as I knew that if he didn’t get the ball as the first threat I needed to push off if they hit the guy out of the back. They were doing this quite a bit and they would block you with that lead runner. I was consciously thinking to chop my feet, so I could quickly react if they went out the back and thinking just don’t engage him because that will leave a gap. I’ve got to stay on him on the hard line and then the man out of the back which we call a ‘gun’. But what they were doing well was that the two front line runners would bump you in the defensive line so you couldn’t then get to the man with the ball out the back. If they’re just carrying the ball and there’s no passing option or threat then you don’t need to think about it. But when there’s a gun option I have to wait, I can’t just hit, if he pulls it out the back I’ve got to get passed the tip option who’s trying to block me and then get to him – which is probably why I miss the tackle. (*UNI Player 8, Game 2*) “Probably quite a bit slower than the others, again because I’ve got to defend the forward, defend the lead which is the hard line and runner coming through, then I have to get to the shadow, which is the running line out the back. Then as soon as the guy out the back passes, then I’ve just got to stay high on the inside. Ideally, if I could stop that play there and then I’d do it but because they played it quite well I knew immediately that I wouldn’t be able to do that. So I looked after him, then had to go and help my man outside of me. What got me was the amount of decisions I had to make in the play, I think I was probably fast on the decisions I made but there was so many decisions I had to make, I found myself recalling doing these things in training and dealing with multiple threats. For us, we always have a principle – ‘always defend the threat’. So defend the ball, if I’m the next man I have to defend him and then I push off.” (*PRO player 1*, Game 1). “I remember thinking that we were stressed and under pressure on the edge [space toward the touch line of the pitch] and I’ve waited on the ‘lead’ too long, tried to get out and just missed the tackle.” (PRO Player 6, Game 2) “I’m on 9 here, in guard 9, 1 and I’m telling myself the same thing, ‘don’t get beat,’ and yeah he went I wasn’t tight enough and ended up getting beaten.” (*UNI Player 8, Game 1*) “I have a specific job here at ‘20’ as when 9 runs I’ll shout to the inside man, and I’d hope he’d cover me better than he actually does here. So as 9 picks I know that he’s my primary focus and then I’m just pushing off, but as he didn’t pass he steps back inside. The whole time I’m in this position I’ve got a role to fulfil, pushed off a little bit as I know there is a threat on the outside, but I know that that is my primary role. I was hoping our ten would step up a little bit and plug that, which stops their 9 being able to dart like that, but in the end as he runs it’s just a decision to try and make the tackle.” (*PRO Player 6, Game 2)*
Offensive situations	Fast-thought	Common language, Defensive picture Tactics Change in expectations & Awareness of action capabilities	“So I had a look up thought that **** is at ten and we’re in what we’d call a lead shape, a lead shadow – which might also be called a wedge, 2/3 etc… and I made that call because of identifying the attacking personnel I had around me.” (*PRO Player 1, Game 1*) “I was initially looking outside me, as I thought that’s where the support would be coming from as we had pre-called that it would be a hit. It was a pre-call pat hit. Because **** should be an option, but once I make the break I look outside straight away, so that’s my initial thought on the pass.” (UNI Player 4, Game 2) “That’s just because with **** I’m thinking he’s going to carry here and when he got stopped I was kind of expecting the ball in an offload position, but when you actually get it I’ve got no idea where I’m going or what I’m doing so I literally just got the ball and reacted to defenders and someone bit on me which like I just went the other way and just tried to run.” (*UNI Player 2, Game 2*) “So here, he doesn’t get tackled as fast as I’m expecting, so my mindset changed, and changed quickly. I don’t want him to get held up so I’m just going to whack him and get him going forward, which made it easier for **** to hit the deck.” (*PRO Player 3*, *Game 1*).

The **** denotes names and terms that have been removed for confidentiality and anonymity.

### Deductive Content Analysis

From the sixteen players who took part, 1896 decisions were verbalised during self-confrontation interviews (UNI: 826; PRO; 1070). Players in the UNI group verbalised 111 *Unstimulated* decisions whereas players in the PRO group verbalised 110 decisions. Both groups of players referenced their actions-for the game most often (UNI = 50%: PRO = 50%), followed by perception of information (UNI = 31.43%; PRO = 22.22%) and a common frame of reference (UNI = 20%; PRO = 17.78%). Knowledge-of (UNI = 7.14%; PRO = 6.67%), and knowledge-in (UNI = 2.86%; PRO = 3.33%) the game were referenced least often.

During the stimulated self-confrontation interview process, 83 and 119 No-thought, 318 and 471 Fast-thought and 314 and 370 Slow-thought decision classifications were made by the UNI and PRO players, respectively. Figure 2 presents the relative frequency of No-, Fast- and Slow-thought decision classifications made within each deductive theme. Chi-squared tests of independence confirmed differences in the frequencies of decision classification for each deductive theme (all *X*^2^ (1, *N* > 137) > 17.52, *p* < 0.01). Observation of Figure 2 shows that for both groups of players Fast-thought decision classification were most frequent within the *perception of information* theme. Fast-thought decision classifications were also prevalent within the *actions-for* the game theme, as were No-thought classifications; Slow-thought classifications were relatively less frequent. Within the *knowledge-of* and *knowledge-in* the game themes, no decisions were classified as No-thought and very few decisions were classified as No-thought within the *common frame of reference* theme. Furthermore, within the *knowledge-of* the game theme there is an observable difference in the relative frequency of Fast- and Slow-thought decisions between the two player groups. A large majority of decisions of UNI players were Slow-thought whereas PRO players show more of a balance of Fast- and Slow-thought decisions. Within the *knowledge-in* the game theme Fast-thought decisions were most prevalent for both player groups. Finally, within the *common frame of reference* theme while Slow-thought decision classifications were most prevalent for both groups of players, this seemed to be particularly the case for UNI players.

### Elementary Units of Meaning

#### Unstimulated Verbalisations

Table 2 demonstrates that players, were able to recall and verbalise their decisions and thought process leading to a decision. These verbalisations were cross-referenced to player’s stimulated references to the same decisions during stage 3 of the data collection procedure. This analysis indicates that player’s often recalled decisions that were later classified as Fast-Thought and Slow-Thought, but not No-thought. Additionally, the units of meaning derived from unstimulated verbalisations were representative of the units abducted from Fast-thought and Slow-thought decision classifications. Player’s often only verbalised their decision making process in relation to an action that was significant in memory following the game (See Table 2).

#### Self-Confrontation Interviews

Table 3 exhibits the deductive and inductive elementary units of meaning that emerged from each deductive theme; *perception of information*, *actions-for*, *knowledge-of, knowledge-in* and a *common frame of reference*.

#### Perception of Information

##### No thought

Players described intuitively responding to *audio* information and having *no time* to think about their decision;

“Just caught the ball without any time to make a decision, felt afterward that I didn’t know what decision to make. Didn’t know whether to pass it, give it to **** out the back. I just carried.” (PRO Player 6, Game 2).

Players described perceiving discrete visual information, with specific reference to; their *opponents*’ *movements, opponents body position, teammates movements* and the *rugby ball*. For both populations’ players verbalise reacting without conscious thought to their opponents’ or teammates movements. For instance,

“Just tackled him, I’ve just stood up and he’s ran into me.” (PRO Player 6, Game 1).

Players also verbalised instances where seeing the ball initiated unconscious responses;

“The ball just kind of pops up, it’s not expected yeah I didn’t know or had a thought about who was around me or what was around me so, it was just catch and carry.” (UNI Player 4, Game 2)

##### Fast thought

For both groups the perception of information is referred to most frequently within Fast-thought classifications (> 34% of responses in both player groups). Eight elementary units of meaning included; *audio* information, *haptic* information, *opponents body positions*, *opponent’s movements*, *teammates movements*, *defensive* and *offensive pictures*, and *time*. Players from both groups refer to their *opponents* and *teammates movements* frequently;

“He’s stepping in, I’ve sort of anticipated him stepping in as well, then I thought about it, but it was definitely a last second thing.” (UNI Player 5, Game 1).

Players also refer to the perception of global information in the form of *offensive* and *defensive pictures*, for example;

“As I’m walking in I’m scanning to see what picture they’re showing me and then I’ll call wherever the space is.” (PRO Player 8, Game 1).

However, PRO players verbalised the perception of discrete information more often than the UNI group, such as their *opponent’s body positions*. The majority of these instances were verbalised during their decision making in the tackle area;

“As the tackle unfolded he started to fall toward the touchline at which point I pulled him and pressed his shoulders and head down toward the touchline. It was fast thought based on feel of, oh we can actually turn the ball over here, so I manipulated his body rather than going for the ball so he’d go out of touch.” (PRO Player 2, Game 1).

##### Slow thought

Units included; *audio* information, *haptic* information, the *defensive* and *offensive* picture, *opponents’ movements*, *teammates movements* and having *time* to perceive. Analysis demonstrates frequent references to more global sources of game information regarding the defensive or offensive picture provided by their opponents;

“The picture changes as we go up, I knew that I could get pressure on him as he didn’t have an option on the outside.” (PRO Player 2, Game 2).

Finally, both populations recall having sufficient *time* to perceive and interpret wider sources of game information, their *opponents’ movements* and/or their *teammates movements* whilst making decisions;

“A lot of time on the ball and a lot of time to compose myself. I was hoping the ball was going to run dead so when it held up I still had a lot of time on the ball so, when I picked it up and turned around I had about 15m to work with and compose myself.” (UNI Player 3, Game 2).

“I’ve got a lot of time. I’m the third guy away from the ruck so instantly I know it’s my job to go at their ball carrier.” (PRO Player 7, Game 1).

In summary, during No-thought verbalisations players recalled perceiving discrete information due to a lack of time to see, hear or feel anything else. Whilst during Fast-thought verbalisations, players referred to the perception of discrete sources of information; such as opponents’ movements or body positions, and global information; such as the defensive picture painted by their opponents to make decisions, whilst the PRO group recounted using discrete information to make decisions more often than the UNI group. Conversely, interpretation of Slow-thought verbalisations indicates that an increase in time offered by particular game situations results in a tendency to focus on global information, such as the offensive or defensive picture provided by their opponents (See Table 3).

#### Actions-for the Game

##### No thought

Player’s described *reacting* unconsciously to game information through their actions. For example, both PRO and UNI players describe similar processes in different game situations;

“It was just an instinctive pass on the inside. I’m under a bit of pressure and there’s my get out.” (PRO Player 1, Game 1).

“I don’t remember what was going on there, I definitely turned it over but it was bit of an instinct thing to just get the ball back.” (UNI Player 4, Game 2).

Less frequently, specific individuals on both teams indicated that *rehearsal* of actions allowed them to act appropriately. For example, the Hooker from the PRO group of players (Player 5) indicated that all of his lineout throws were No-thought;

“That’s just me following my process – we’ve rehearsed where he should be so I just throw it.” (Game 1).

##### Fast thought

Players describe moments of self-awareness whilst making decisions, however PRO players refer to this more frequently than the UNI group. For example;

“I’m sprinting thinking I’m going to compete in the air, but then in looking at the flight of the ball and I know that it’s too far in front of me and I know I’m not going to get to it.” (PRO Player 1, Game 2).

Players verbalisations regarding their actions-for the game often referred to having knowledge of their *intentions* to achieve a specific outcome. Players from both populations consistently refer to “knowing,” “having to,” or “needing to” act in a specific way to solve the problem in front of them. For example;

“I just dropped my shoulder to try and get through. I’m first to the ruck, my decision is always to clear any threat.” (PRO Player 3, Game 1).

##### Slow thought

Slow-thought verbalisations regarding players actions-for the game were more infrequent than No-Thought and Fast-thought classifications, but often recounted clear thought processes during decision making, irrespective of whether the outcome was successful or not. For example, UNI and PRO players recall being self-aware during their decision making;

“Because we ran a kick chase line, he was my opposite number and I nominated him, as my man, tracked him.” (UNI Player 2, Game 2)

“But instead I made him step back on my inside shoulder and he’s actually quite a good stepper for a big bloke, then I just thought I need to lunge at his ankles here to get him.” (PRO Player 6, Game 2)

Players also described being more aware of the capabilities of their *teammates* within their verbalisations of slow thought decisions;

“The reason being was ****, the prop, told me to run near him and he does it in training it’s like a proper old man’s trick just like nudging someone. So like, as I caught it I heard that and I thought s**t where’s that coming from. I looked up and saw him.” (PRO Player 5, Game 2).

Within Slow-thought decisions, most verbalisations of player’s actions-for the game tended to be dependent on *intentions* derived from Knowledge-of what action was appropriate for the game situation specifically.

To summarise, No-thought verbalisations referred exclusively to players unconscious reaction to perceptual information, whilst individual player’s made reference to the importance of rehearsal. Interpretations of the Fast-thought data suggests that their intentions, shaped by their perception of information, guided resultant action in time-pressured situations. Whilst, Slow-thought classifications indicated that action-for the game depend on a player’s, self-awareness, intentions and an awareness of their teammates action capabilities (See Table 3).

#### Knowledge-of the Game

##### Fast thought

Within fast thought classifications, players occasionally referred to their knowledge-of their *opponents*, *roles and responsibilities, self-awareness* and *tactics*. However, the results indicate that PRO players verbalised these units more often. Furthermore, they referred to tactical *self-awareness an*d knowledge-of *teammates* when coming to fast thought decisions;

“He’s a very good off loader, so I knew that his first instinct would be to give the ball, so I jumped at the chance haha.” (PRO Player 5, Game 2)

##### Slow thought

Players referred to consciously using knowledge-of their; *opponents*, *roles and responsibilities*, *self-awareness, tactics* and their *teammates* whilst making decisions (>23% of responses). The main difference between the groups verbalisations rested on the PRO players making reference to knowledge-of their opponents to inform their decision making more frequently;

“I looked up here and saw their 7 **** was in front of me, who’s their most effective jackler, so I had time to think if he makes the tackle there’s less chance of him then making the turnover. Just thought, get him to make the tackle, stay tight.” (PRO Player 5, Game 1).

The vast majority of slow thought verbalisations regarding players knowledge-of the game make reference to the importance of executing specific *roles and responsibilities* that are essential within particular game situations;

“I have a specific job here. So as 9 picks I know that he’s my primary focus and then I’m just pushing off, but as he didn’t pass he steps back inside. The whole time I’m in this position I’ve got a role to fulfil.” (PRO Player 4, Game 2).

On numerous occasions both groups refer to perceiving and acting based on their tactical approach to the game;

“That’s our game plan, to play the bottom corners and that’s why I looked. Yeah that is the reason why I’ve looked. Erm, I’ve just kicked it because he was a bit high in the line.” (UNI Player 2, Game 1).

Thus, knowledge-of the game was rarely made reference to during UNI and PRO players Fast-thought verbalisations but on occasion, players referred to the team’s tactics and the roles and responsibilities that guided their decision making. The Professional players made reference to tactical self-awareness and using knowledge-of their teammates during Fast-thought classifications. Analysis of Slow-thought responses indicate that when players had time, they often referred to their knowledge-of tactics, roles and responsibilities as navigators of perceptual search and choice of action. In addition, the Professional players knowledge-of the game extended to knowledge of their opponents’ tendencies, tactics and behaviour (See Table 3). No reference to knowledge-of the game was made during No-thought verbalisations.

#### Knowledge-in the Game

##### Fast thought

Player’s referred to their knowledge-in the game most often during Fast-thought decision classifications. In coding these verbalisations into elementary units of meaning, players across both groups described that their expectations of the situation were regularly unsatisfied. Verbalisations indicate that players had to adapt quickly to *changes in expectations* regarding game situations. UNI players referred to adapting to *changes in expectations* frequently (>67%) during verbalisations of Fast-thought decisions, indicating a tendency to form unreliable expectations within game situations.

“I’m pretty sure I was supposed to run a 3 on that one, but I ended up getting the ball unexpectedly.” (UNI Player 4, Game 1).

In addition to changes in expectations, players from both groups describe the impact of *game context* on their decision making in Fast-thought instances;

“That was probably happening a bit quicker and like a said probably a bit of panic. The context of the game as well as they were coming back into it as well.” (UNI Player 1, Game 2).

Finally, UNI and PRO players refer to dealing with legitimate *risks* or *threats* to their offensive or defensive integrity. PRO Player 5 describes an instance where he’d noticed an opposing player, who he knew to be strong at competing for the ball, in close proximity as he was tackled;

“Then as I land, I’m more conscious of my groundwork because of the threat of ****, so I’m kicking my hips and rolling, so he just didn’t have a target to get on the ball.” (Game 1).

##### Slow thought

Players verbalised having to update their knowledge-in the game less often during Slow-thought decision classifications. However, units of elementary meaning demonstrate that when they did it coincided with similar themes; *changes in expectations*, the *game context* and realistic *threats* or *risks* to offensive or defensive integrity. However, PRO players made reference to the *game context* more often than the UNI players;

“Again this was a case of awareness on the field. I’m not wanting to take the ball to get personal glory out of what is a no win situation so told him to carry again” (PRO Player 1, Game 2).

Player’s form expectations of situations from their perception of the game situation and their knowledge-of the game, however these expectations are frequently unmet (See Table 3). Furthermore, following recognition of this, players tended to take the first option available to them. This is most evident within verbalisations of Fast-thought decision classifications where knowledge-in the game is referred to consistently. During Slow-thought verbalisations, the PRO players made reference to the use of game context, such as the score, the time left in the game and their awareness of offensive or defensive momentum more often than the UNI group. No reference to knowledge-in the game was made during No-thought verbalisations.

#### Common Frame of Reference

##### No thought

On five occasions PRO players referred to *common language* and *tactical rules* initiating action without conscious thought. For example, PRO Player 3 verbalised having “hit the ruck on autopilot” (Game 2) without thinking about it. One UNI player referred to the use of common language whilst verbalising a no thought decision.

##### Fast thought

Players often referred to the use of *tactical rules* to come to decisions within their Fast-thought classifications. For example, multiple players from the Professional group refer to a “tackler plus one” rule instigated by their coaches. Players often describe having to make a rapid judgment as to whether it is their responsibility to join the original tackler in order to slow the ball down;

“We have a tackler + 1 rule, so if you are going to go in there, be effective and slow the ball down.” (PRO Player 7, Game 1).

Similarly, UNI Player 8 indicated that they too follow tactical rules, for instance when their scrum half is caught up in the ruck situation, they resort to a typical course of action;

“That’s a fast thought, if 9 doesn’t go in we bullet. If 9 goes into the ruck it’s a pick and go, simple.” (Game 1).

These rules tend to coincide with the use of shared terminologies that are included in a wider common language between teammates and coaches. For the PRO players, 13 terms were verbalised that captured tactical rule’s and plays, whilst UNI players verbalised 16. Player’s recounted hearing, or using these terms to guide the decisions and actions of themselves and others in time-pressured situations;

“I was screaming 3 and because I was coming around the corner, I had a bit more depth. When I got outside the second last defender, it was essentially a 3 vs 1 on the edge” (UNI Player 4, Game 2).

In addition to common language, players also described general communication between them and their teammates in coming to decisions;

“**** told me just to carry so I just carried” (UNI Player 6, Game 2).

##### Slow thought

A common frame of reference was referred to frequently during verbalisations of Slow-thought classifications (both groups > 30%). Elementary units of meaning indicate a clear role of verbal and non-verbal communication between players when making decisions. These were coded into; a shared *common language*; *general communication* between teammates; and *tactical rules*. Both groups describe shared understanding of a common language that influences players collective and individual decision making by guiding perception to more relevant game information, informing teammates of information players are unable to perceive visually, and shaping clear intentions of how they should act given the circumstances. For example;

“He then brings us in for a “truck” which is a slow decision to carry the ball into contact. He then say’s “apple,” which is a box kick so I’ve got to get wide for the kick chase.” (PRO Player 5, Game 2).

“That was just us running an addidas, **** was running a hard line and then one of their lads jumped out and that meant **** was the only option.” (UNI Player 4, Game 2).

These terms also actuated players decisions to execute rehearsed *tactical rules*. For instance;

“The call reminds me of my course of action after I’ve thrown the ball in. At 99% of mauls my role is to get the ball at the back, it’s just my job.” (Professional Player 3, Game 1).

Finally, players recall the use of general communication between them and their teammates whilst making collective decisions;

“Our aim is generally to get the ball to the edge within our team pattern so there was no point me carrying it because we’d have had a weaker clear if I’d carry it, so I told him to carry it and talked him through carrying it and then knew I was hitting the ruck straight away.” (PRO Player 1, Game 1).

To summarise these findings, players refer to a common frame of reference across all decision classifications (See Table 3). Both UNI and PRO players refer to the use of a common language that is made up of specific terminologies that guide players collective perception of information and actions-for the game within their decision making processes. These terms house tactical rules for players that are included in a wider common language, resulting in planned and prescribed actions and movements. Such terms are referred to most often during Slow-thought verbalisations, whilst occasionally in Fast-thought and on a handful of occasions during No-thought classifications.

#### Game Moments

Table 4 highlights the perceptual information, cognitive mechanisms and tactical strategies verbalised by players in specific game situations, most specifically, the lineout, the scrum, defensive situations and offensive situations.

##### Lineout

Individual players from each team were given sole responsibility for running and calling their lineout. Players used a common language shared between their forward unit to initiate the movement coordination of a throw by the hooker, lift by two supporting players and jump and catch by the jumper. Players described having a menu of tactical rules, housed under particular shared terminologies, that were selected before the game after watching and identifying how their opponents tended to behave. For Slow-thought verbalisations, UNI Player 8 and PRO Player 7, both verbalise how they would enter the lineout with a pre-call, which they would immediately execute if their opponents presented the space to them. If not, they referenced that a Fast-thought was required to perceive the defensive picture rapidly before calling a second term (or body movement) that teammates would respond too. Both players consistently refer to the importance of having time to perceive and assess the initial defensive picture before adapting.

##### Scrum

Scrum situations were exclusively classified as Slow-thought where players referred to the execution of a “checklist” in response to haptic game information (See Table 4). The data implies that this checklist is formed from knowledge-of what action is appropriate given the feel of their scrummaging position. Alongside this, both teams had a common language that would initiate how they intended to approach the scrum, that is, being aggressive or being more conservative.

##### Defensive situations

Defensive situations ebbed and flowed between open phase play, and tackle situations. For both instances, players verbalised that tactical rules often dictated what the players role, responsibility and intentions should look like. But, on multiple occasion’s the players described being consciously aware of too many pieces of game information with too little time, resulting in paralysis by analysis and missed tackles. The data implies that the tactical rules drove players to focus less on the perceptual information provided by opponents and more on rules associated their own movement behaviour. For example, in open phase play, players who played the position of centre, were required to abide by the rule of *waiting* on the threat of a hard line of running, before moving toward the other option which more often than other positions, which often resulted in missed tackles. Similarly, forward verbalised their role and responsibility of their positioning next to the ruck situation, their position demanded a role (terminology) and responsibility (which opponent). Player’s verbalised feeling internalised pressure in these moments which resulted in numerous missed tackles.

##### Offensive situations

Offensive situations also shifted between open phase play (carrying, passing and kicking) and tackle situations (tackle, ruck, maul). Nonetheless, both teams often refer to a framework of play that drove their positioning and intentions. Slow-thought decisions often coincided with moments of open phase play, for instance players verbalised calling a tactical play housed under a common term to pre-plan their action response to the defensive picture provided by their opponent (See Table 4). No-thought and Fast-thought decisions coincided with moments where players initial assessment of situational probability was unsatisfied resulting in a need to reassess or react to game information. These moments were often verbalised during local interactions between attackers and defenders and within the tackle situation and ruck.

## Discussion

Sixteen adult rugby players conducted three stages of data collection within 48 h of two competitive fixtures to: (i) consider how game situations influence players *perception of information*; (ii) to consider how game situations influence the application of cognitive mechanisms whilst making decisions; and (iii) to identify the influence of tactics and/or strategy on player decision making. (Raab, 2003; Bar-Eli et al., 2011; Araújo and Bourbousson, 2016; Birch et al., 2019; Bourbousson et al., 2019; Ashford et al., 2021). Players were required to verbalise their lived decision making experience during self-confrontation interviews, which were framed along a continuum of No-thought, Fast-thought and Slow-thought (Varela and Shear, 1999; Macquet, 2009; Bourbousson et al., 2010; Macquet and Kragba, 2015; Johnston and Morrison, 2016). Verbalisations were subject to two stages of analysis including; a deductive content analysis in relation to Ashford et al.’s (2021) unified framework for decision making in team invasion sports; and the deductive and inductive collation of responses into elementary units of meaning (Thereau, 2003; Macquet and Kragba, 2015; Gleeson and Kelly, 2020). As anticipated, our interpretation of the data indicates that game situations have a clear impact on players perception of information and the cognitive mechanisms used, or not used, within the decision making process. Furthermore, tactics and strategy seem to have a significant impact on player decision making.

### Perception of Information

Verbalisations of fast and frugal decisions coincide with references to the perception of global (Lex et al., 2015; Johnston and Morrison, 2016), and more discrete (Jackson et al., 2006; Johnston and Morrison, 2016) information. Our findings suggest that players perceive game information on a global scale; such as the width of the defensive line, identifying space in backfield or identifying advantages and disadvantages between attacking and defensive numbers, as *pictures* that invite particular courses of action (Lex et al., 2015). This is comparable to the findings of Johnston and Morrison (2016) who compared perceptual strategies of skilled and less-skilled rugby league players. They suggested that skilled players perceive cues of a global nature whilst lesser able players focus more on discrete local information, such as the body positioning of opponent’s (Johnston and Morrison, 2016). In contrast, our findings demonstrate that both groups of players perceived discrete information as frequently as global information when making decisions classified as Fast-thought. Players make reference to their opponent’s body positions, opponents’ movements and teammates movements as actuators of the decision making process (Passos et al., 2008; Correia et al., 2012, 2013). Interestingly, the perception of global, or discrete information seems dependent on the situational context placed on the decision maker. The perception of global information, such as the width of the defensive line, often coincided with moments where players were waiting for the game to come to them in open phase play, such as a backline waiting to receive the ball at the lineout (Macquet and Kragba, 2015). Instead, recall of discrete information during player verbalisations tended to correspond with tackle situations, for example, a one vs one situation between a ball carrier and a tackler (Jackson et al., 2006; Correia et al., 2012, 2013).

The analysis of No-thought and Slow-thought verbalisations demonstrates that players are presented with variances in time available to make decisions during a game of rugby union (Raab, 2003). The No-thought data implies that players often refer to having no time to perceive game information, whilst during Slow-thought verbalisations both groups often allude to having increased time, providing an opportunity to view a global array of visual perceptual information. These findings provide further evidence to the idea that perception is task dependent as semi-elite player’s verbal descriptions change in line with different game situations (Lenzen et al., 2009; Roca et al., 2011, 2012, 2013). In rugby union, the rules dictate that a contest for possession of the ball occurs every time attacking and defensive players meet (Ashford et al., 2021). Subsequently, our findings demonstrate that players who are involved in the contest for possession, such as the ball carrier, tackler and supporting players, have minimal time to perceive and act on game information. In such time pressured situations, a *psychological refractory period* may occur, where players are unable to perceive a secondary source of game information (Araújo et al., 2004; Maslovat et al., 2013). Therefore, players must rely on the initial perception of discrete information in the local array, such as their opponent’s movements and body positions (Correia et al., 2013). Conversely, players who are not involved in the contest, are given time to perceive a global array of offensive or defensive *pictures* offered by their opponents, to communicate and collectively execute a decision for the next phase of play (Johnston and Morrison, 2016).

Players communicated the use of different perceptual sources, presenting visual (Johnston and Morrison, 2016), acoustic (Davids et al., 2008) and haptic information (Lenzen et al., 2009), whose use is predicated on the position they play and the game situation (Raab, 2003). Whilst the majority of decisions within open play are actuated by global or discrete visual information, players also perceive acoustic information from teammates, especially in backline positions. Furthermore, in particular game situations such as the scrum (See Table 2) players mostly rely on the perception of haptic information to make decisions. For instance, Props from the UNI and PRO playing groups indicate that the conscious awareness of haptic information; such as the strength of their physical scrummaging position and their bind between themselves, their teammates and their opponents actuates their decision making process. Consequently, inferences suggest that rugby union coaches should guide players to global, discrete, audio or haptic perceptual information based on the time available to a player within particular game situations.

### Actions-for the Game

Analysis of verbalisations making reference to player’s actions-for the game suggest that UNI and PRO players perception of information is guided by their physical (Passos et al., 2012) and technical (Paterson et al., 2013) capability to act (Ashford et al., 2021). Within Unstimulated, Fast-thought and Slow-thought data, players often recount being consciously aware of their physical and technical capabilities when perceiving and acting on game information. In contrast, analysis of No-thought verbalisations often unearthed tendencies to refer to an self-organised response, which in the majority, still resulted in positive outcomes. Players from both teams also rarely verbalised decisions where they had misjudged perceptual information. This may present evidence that both UNI and PRO players physical and technical capabilities to act are coupled with highly salient game information (Davids et al., 2008; Passos et al., 2008; Correia et al., 2013). Thus, players perception of information may, at times, depend on a refined connection to their technical and physical capability (Jackson et al., 2006; Esteves et al., 2011; Paterson et al., 2013).

For the majority of No-thought classifications, players could not remember nor verbalise how or why they came to act in such a way. Instead, they referred to reacting without thinking or based on a feel for the situation. This may suggest that within these moments, the driving mechanism of the players decision making process is a successful (or unsuccessful) forming of a direct online relationship between game information and their technical and/or physical capabilities without need for memory representation (Cappuccio, 2015; Wilson et al., 2018; Raab and Araújo, 2019). It is important to note however, that such verbalisations of implicit self-organisation were rare, yet this may be due to the methods adopted within this study. Furthermore, despite players indicating that they’d decided based on feel or through reaction, they often impressed wider knowledge of what was required to satisfy the situation within their verbalisations. Subsequently, these findings from No-thought classifications may be interpreted in two ways; first, as evidence for a direct and enactive relationship between perception and action within the decision making process (Cappuccio, 2015) or second, evidence for Changeux’s (2009) view, that even in situations where players are denied access to conscious thought, neural embodiments of meaning stored as representations allow for players to satisfy the demands of the game situation. Analysis of the data suggests that players had an instinct of what was right given the game situation, suggesting that Changeux’s (2009) view may be better placed to explain these findings. Additionally, whilst verbalising a particular No-thought classification, a PRO player made reference to the importance of rehearsing his lineout throw in hope of removing conscious thought from his decision making process. Interestingly, our data suggests that despite these preferences, the decision of where to throw was still cognitively driven through memory representations, as the player refers to having to listen, interpret and act on a common term which initiated where the ball needed to be thrown (McPherson and Vickers, 2004; Richards et al., 2012, 2017). Instead, the player’s desire to remove conscious thought from the decision, was in reference to the execution of the throw itself, perhaps aware of the need to free resources for the decision making process.

In contrast, elementary units of meaning abducted from Fast-thought and Slow-thought classifications suggest that player’s actions are often driven by situation specific intentions. However, these intentions are determined by a player’s deep declarative and procedural understanding of what, how and why to act on given game information (Anderson, 1982; Macquet and Fleurance, 2007; Lenzen et al., 2009). This challenges a widely accepted contemporary view that the coupling of a player’s perception and action are bound directly (Passos et al., 2008; Raab and Araújo, 2019). Instead, the data of the current study suggests that perception, whilst coupled and refined with salient game information, is often predicated by the retrieval of conscious memory representations that initiate what action is selected and why (Lenzen et al., 2009; Macquet and Kragba, 2015; Johnston and Morrison, 2016). Furthermore, players described being self-aware of their technical and physical capability during the decision making process. Interestingly, these moments demonstrate a tendency to engage in meta-cognitive processes in fast paced game situations, where players consciously assess whether they possess the technical, and/or physical capability to influence the outcome positively (Price et al., 2019, 2020). Finally, the interpretation of data suggests that statements that describe the relationship between perception and action as one directly connected, for instance that players must “perceive to act and act to perceive” (Fajen et al., 2008; Silva et al., 2013), should be extended to account for the interceding mechanism of memory representation (McPherson and Vickers, 2004). That is, in order to perceive to act and act to perceive (bottom-up), one must possess the knowledge of when, where and why to perceive and act accordingly (top-down; Anderson, 1982; Ashford et al., 2021).

### Knowledge-of the Game

The influence of game situations are clearly demonstrated in our findings of the deductive content analysis. No references were made to knowledge-of or knowledge-in the game during verbalisations of No-thought classifications (Raab, 2003; Bar-Eli et al., 2011), whilst reference to the use of knowledge-of the game was verbalised only on occasion during Fast-thought classifications. Instead knowledge-of the game was predominately recounted during Slow-thought decision classifications where players were presented with more time and an increased number of options to select from. Within these instances, player’s share how their knowledge-of opponents, tactics, roles and responsibilities, self-awareness and teammates all influenced their decision making process (Gréhaigne et al., 1999, 2005; Richards et al., 2012; Macquet and Kragba, 2015). Our findings suggest that in time rich situations, memory representations, stored as task-specific declarative knowledge, are the key connecting variable between the perception of game information and the decision to act in a certain way (Anderson, 1982; Lex et al., 2015; Johnston and Morrison, 2016; Birch et al., 2019). Thus, when players have time, their search for game information and their selection of a relevant capability to act are both guided by knowledge-of their team’s tactical approach (Mulligan et al., 2012; Richards et al., 2017). This presents clear evidence that rugby players store and use memory representations to perceive, interpret and act on tactics, roles and responsibilities successfully (Poplu et al., 2008; Johnston and Morrison, 2016; Schulz, 2018; Birch et al., 2019; Raab and Araújo, 2019).

The elementary units of meaning comprised from player’s knowledge-of the game all relate to the successful execution of pre-planned intentions for performance (McPherson and Vickers, 2004; MacMahon and McPherson, 2009). Our data demonstrates that improved memory representations allow players to form suitable plans of action, through tactics, roles and responsibilities, which enhance their capability to effectively perceive and operate in dynamic game environments (McPherson and Vickers, 2004; Ashford et al., 2021). Such plans of action guide perceptual strategies, the retrieval of memory representations and subsequent action, for example, knowing when, where and why to kick the ball up field in order to relieve defensive pressure (McPherson and Vickers, 2004; Macquet and Fleurance, 2007). These extended to the recall of knowledge-of opponent’s tactics or individual tendencies to make effective decisions during Fast-thought and Slow-thought verbalisations. Interestingly, research has suggested that being consciously aware of movement tendencies can be detrimental for anticipatory performance (Memmert and Furley, 2007; Jackson et al., 2020). However, such research has focussed on player’s ability to detect deceptive intent in one vs one situations (Jackson et al., 2006, 2020). Whereas, in the context of this study, references made to knowledge-of opponents by players were often made in association to game situations where players had sufficient time to make decisions, such as the lineout or scrum. Consequently, we encourage coaches and players to develop knowledge-of opponent’s tactical tendencies to inform player decision making.

### Knowledge-in the Game

Interpretation of these findings suggest that reference to knowledge-in the game was made predominately during Fast-thought verbalisations (Raab and Gigerenzer, 2015; Hafenbrädl et al., 2016). Interestingly, players describe that they tend to form expectations of what will happen based on their assessment of game information (Macquet, 2009; Eccles and Tran, 2012). In the first instance, this demonstrates that players attempt to make sense of game information and engage in an assessment of situational probability to successfully anticipate an outcome (Eccles and Tran, 2012; Macquet and Kragba, 2015; Loffing and Cañal-Bruland, 2017; Helm et al., 2020). However, the findings suggest that players were often inaccurate when calculating situational probability from their assessments of game information. Players described having to update their perception of the situation as game information altered before them (McPherson and Vickers, 2004). Macquet and Fleurance (2007), Macquet (2009) would suggest that at this moment, players would deem this information atypical and be required to process available information in relation to formed mental representations to generate an appropriate response. Interpretation of these findings suggest that, in these instances, players took the first viable option presented to them to quickly satisfy the situation and remove risk (Johnson and Raab, 2003; Raab and Gigerenzer, 2015). Interestingly, UNI players were required to update and adapt to game incidents more often than the PRO playing group. This is likely due to an over reliance of the UNI playing group on sticking to tactical intentions (Passos et al., 2008; Bourbousson et al., 2011); and PRO players increased perceptual capability to form an accurate assessment of situational probability (Eccles and Tran, 2012; Loffing and Cañal-Bruland, 2017; Helm et al., 2020). Furthermore, Den Hartigh et al. (2018) Skill Theory may offer some insight to these findings. They compared the recall ability of selected and non-selected soccer players to consider the complexity and declarative knowledge base within verbalisations. Their findings demonstrated that declarative knowledge was not dependent of complexity or competitive level, suggesting a dissociation exists between players declarative knowledge and knowledge-in-action. This disassociation may explain the inaccuracies in both populations to successfully assess the situational probability of game situations (Den Hartigh et al., 2018).

Players recognised legitimate risks or threats to their attacking or defensive integrity within Fast and Slow-thought classifications, for instance, losing possession of the ball to their opponents or conceding a try. Macquet and Kragba (2015) found that elite female basketball player’s make sense of game information to make decisions in two steps: (i) an assessment of the situation and (ii) anticipation of situations as they develop to manage risk. Similarly, our findings demonstrate that a clear relationship exists between player’s sense-making and decision making, as player’s knowledge-of the game seems to guide their perceptual assessment of the situation (Del Missier et al., 2010; Macquet and Kragba, 2015). Identifying risks and threats coincided with sudden perceptual acknowledgement of an opponent’s capability to disrupt momentum. Furthermore, these verbalisations referenced incidents that occurred in fast paced situations that disrupt player’s situational probability, such as an opposing player attempting to steal the ball in a ruck situation, (Ashford et al., 2021). Raab and Gigerenzer (2015) identify that when humans are required to make decisions in time pressured situations they tend to rely on heuristics, that is, bias of what has worked before (Jackson et al., 2006). Consequently, when player’s perceived legitimate risks or threats, they often used deeper declarative knowledge-of the game to update their knowledge-in the game as they took the first option that presented itself (Raab and Laborde, 2011; Raab and Gigerenzer, 2015).

The influence of game context during player decision making was verbalised by the PRO group more frequently than the UNI group (González Víllora et al., 2013; Light et al., 2014). Game context has been explored frequently in decision making research, referred to as contextual priors (Mann et al., 2014; Cañal-Bruland and Mann, 2015; Levi and Jackson, 2018; Broadbent et al., 2019). For instance, Lenzen et al. (2009) indicated that verbalisations derived from self-confrontation interviews with handball players demonstrated that the score, players involved, and match difficulty all influence the decision making process. Johnson and Raab (2003) found that soccer players think about their scoring potential (i.e., penetrate the opponent’s defensive line and score) and their safety potential (i.e., losing the ball or conceding) as they progress throughout the game. Relatedly, Levi and Jackson (2018) indicated that player’s extract dynamic contextual priors from game information that effects their decision making process. Dynamic contextual priors include the score status, feelings of momentum, assessment of personal performance and external instruction from coaches or teammates (Levi and Jackson, 2018; Gredin et al., 2019). Our findings demonstrate regular moments where the PRO group of players reflected on their offensive and/or defensive *momentum* when making decisions. On occasion, descriptions of momentum led to the suggestion that players felt levels of situational favourableness, where the game felt completely in their control (Levi and Jackson, 2018). In contrast, the UNI group only make reference to game context in a state of panic when the opposing team came back into the game, demonstrating negative feelings of momentum. Offensive and defensive momentum was often used by PRO players to make informed assessments of risk associated within their decision making (González Víllora et al., 2013; Light et al., 2014; Macquet and Kragba, 2015).

### Common Frame of Reference

Player’s common frame of reference was verbalised frequently within Slow-thought decision classifications. Our findings present evidence that both teams operate, coordinate and communicate their decision making based on a shared mental model of performance (See Table 2, Offensive situations, scrum & lineout; Richards et al., 2012, 2017; Tee et al., 2018). Players referred to game moments where the profound use of a common language had many purposes during individual and collective decision making (Richards et al., 2017). These included perceptual strategies such as; the guidance of teammates perception; to inform teammates of perceptual information they could not visually perceive; to allow players to make sense of game information; to assess situational probability; to identify threats and/or risks; and finally, to notice sudden changes in expectations (Richards et al., 2012; Ashford et al., 2021). Furthermore, our data suggests that a common language was the key mechanism upholding each team’s tactical “framework” they were working toward (McKay and O’Connor, 2018). Consequently, our findings suggest that a shared common language not only guides the perception of players, but also initiates their decisions and actions in an intentional pre-planned fashion. Specific common terms often demanded the execution of particular tactical rules, roles and responsibilities by both groups of players’ (McKay and O’Connor, 2018). Thus, the deep declarative understanding of a shared common language between players, connects their *perception of information* to their individual and collective *actions-for the game*, *knowledge-of the game* and *knowledge-in the game* (Ashford et al., 2021). In turn, the consistencies in the data raise a clear question regarding the current theoretical differentiation between shared mental models (Richards et al., 2012, 2017) and shared affordances (Silva et al., 2013) for team coordination. In rugby union, they are, in fact dependent on a deep declarative understanding of teammates capabilities and a clear shared common language (Richards et al., 2012, 2017). Balagué et al. (2019) suggests that coaches should imprint strategy with their player’s, which guides player’s attention to perceive affordances. Subsequently, in relation to the data demonstrated in this study, shared affordances are a likely byproduct of tactical shared mental models (Richards and Collins, 2020).

Interpretation of our data suggests that within key moments (Tee et al., 2018), players used tactical rules, housed within common terms, as either beneficial or detrimental for their decision making performance (See Table 2 for raw data examples). Raab (2003) suggested that the application of decision making processes at an individual level may depend on the cognitive complexity of different game situations. More specifically, those who had developed explicit *knowledge-of* the task were advantaged when cognitive complexity was high, whereas those who had developed *actions-for* rather than *knowledge-of* the task (i.e., implicit learning) were advantaged when cognitive complexity was low (Raab, 2003). However, our data suggests that player’s regarded *time* as the key dependent variable for the application of decision making processes over that of cognitive complexity (Raab, 2003). Game moments such as the lineout, scrum and open phase play offer player’s more time to make decisions and within these moments player’s verbalised the perception of global information, such as the opponents defensive picture (Johnston and Morrison, 2016), the communication of a common term (Richards et al., 2017) which actuated the coordinated execution of tactical rules, roles and responsibilities for all players involved (Del Missier et al., 2010; McKay and O’Connor, 2018). In contrast, when player’s verbalised having limited time, such as dealing with a threat at the breakdown, players recalled perceiving discrete information, such as their opponents body position (Macquet and Kragba, 2015) and tended to take the first option presented to them (Raab and Laborde, 2011; Raab and Gigerenzer, 2015).

Raw data examples emerging through self-confrontation data indicates that players errors in judgement tended to materialise when a misalignment occurred between the time available to a player and their application of a cognitive mechanism (See Table 2, Defensive situations). Verbalisations regarding decisions in specific defensive situations highlight that both groups of players followed a shared tactical rule, which internalised the accepted responsibility to wait for one opponents’ movement to finish before perceiving the next. Frequently, these situations resulted in missed tackles by players as their decision to perceive the second attacker came too late. Here, the data presents that coaches have employed a top-down shared mental model to guide player’s perception and action through set roles and responsibilities in specific game situations (Richards et al., 2012, 2017). However, interpretation of our data suggests that players have on occasion become blinded or paralysed by an over-reliance on a specific memory representation that dictated their behaviour rather, than behaving in response to game perception (Passos et al., 2008; Correia et al., 2013). Players recalled being unable to anticipate the deceptive movements of their opponents (Jackson et al., 2006), unable to make sense of information to identify legitimate attacking threats (Macquet and Kragba, 2015) and unable to adapt quickly enough in response to their opponent’s movements. Consequently, whilst player verbalisations suggest that such decisions are still cognitively driven, in such incidents, a top-down shared mental model can be overbearing if too prescriptive and controlling of player behaviour (Steiner et al., 2017; Ribeiro et al., 2019a, b).

## Conclusion of the Findings

The aims of this study were three-fold; (i) to consider how game situations influence players *perception of information*; (ii) to consider how game situations influence the application of cognitive mechanisms whilst making decisions; and (iii) to identify the influence of tactics and/or strategy on player decision making. Players perception of game information, was in large, dependent on the task or game situation (Roca et al., 2011, 2013). When players had time, they often verbalised global information, whilst as time decreased their perception of information became more discrete (Johnston and Morrison, 2016; Basevitch et al., 2019). The application and use of cognitive mechanisms varied from situation to situation, however the majority of player verbalisations were found to be cognitively driven by memory representations stored as knowledge-of and knowledge-in the game (McPherson and Vickers, 2004; Changeux, 2009; Johnston and Morrison, 2016; Richards et al., 2017). Memory representations were often verbalised as the connection between the perceptual search for information and an ideal action response, thus, UNI and PRO rugby players often formed plans of action in regard to what to look for, hear or feel (Macquet, 2009; Lex et al., 2015). Subsequently, if player’s initial assessment of situational probability was accurate, players described having time to consider options to come to an appropriate decision (Macquet and Kragba, 2015). Finally, players consistently verbalised the role of a shared common language which implies that players coordinate perception and action by communicating. This tactical focus suggests that players are required to interpret verbal terminologies between teammates, which identifies the existence and use of shared internal mental representations (McPherson and Vickers, 2004; Richards and Collins, 2020).

Conversely, player decision making was frequently verbalised during Fast-thought decision classifications, where player’s initial assessments of game situations were inaccurate. Players response to these incidents often coincided with a rapid ability to make sense of game information (Macquet and Kragba, 2015) and a heightened capability to satisfy the situation by taking the first option available to them (Raab and Gigerenzer, 2015). In contrast, verbalisations of No-thought decisions occasionally presented evidence of sub conscious decision making made through feel or reaction. These findings were interpreted in two ways, one as evidence of a direct relationship between perception and action, where players self-organised their behaviour implicitly in response to game information without need for memory representations (Raab and Araújo, 2019). Two, as evidence for neural embodiments of meaning, where representations are formed from a network of neurons that fire in synchronicity following the perception of information (Changeux, 2009). Given the nature of player descriptions the evidence seems to suggest that representations still drive what the player needs to achieve in the decision making process. Despite these interpretations, such descriptions were infrequent and coincided with moments where players had a split second to react to game information (less than 200 decisions across both populations). Nonetheless, if players are required to update, diagnose or self-organise to game information, these incidents must be accounted for in representative training environments (Passos et al., 2008; Raab and Laborde, 2011).

Raab et al. (2019), suggested that the player’s perspective must be considered when seeking to understand cognitive mechanisms and decision making processes (Raab et al., 2019; Gleeson and Kelly, 2020). Throughout the study there has been an attempt to mitigate the fallibility of verbalisation methods which included framing self-confrontation interviews on a continuum of No/Fast/Slow-Thought. Furthermore, the collection of unstimulated data facilitated cross-reference to player’s responses to self-confrontation elicitation questions. Despite these, the limitations of this approach are unavoidable regarding the reliability of stimulated recall to explore player decision making. It is also important that the limited references made by players to implicit, self-organised decision making processes are a likely consequence of this approach. Thus, it is suggested that future study in this area looks to integrate experimental and ecological task design alongside self-confrontation interviews adopted in this study. That way, the limitations of both methodological approaches can be considered to enhance the trustworthiness of the findings.

### Practical Implications

The data presents that player decision making in rugby union may best be understood by considering the context in which the decision is made (Simon, 1956). Whether implicit, explicit, or through the presence or absence of cognitive mechanisms, player decision making occurs relative to its environment within a game situation (Todd and Gigerenzer, 2001; Gigerenzer and Selten, 2002; Raab, 2003). Recently, Morgan et al. (2020) have explored coaches’ perceptions of decision making in rugby union and through semi-structured interviews indicated that the coaches who participated stressed the importance of a balance between structure and chaos when supporting the development of their players’ decision making. They suggest that deliberate coaching strategies, such as off-field video analysis, game-plans and strategy were as equally important as questioning or the use of game based representative scenarios. Practically therefore, in relation to the findings from this study, we suggest that coaches adopt two types of decision making training environments. Firstly, the use of intentional decision making training (explicit learning) should be adopted to develop cognitive decisions where players know what information to search for and what action is appropriate to satisfy the game information (Bar-Eli et al., 2011). This may include explicit slow deliberate sense making activities such as; video analysis sessions, previews and team debriefs (Richards et al., 2012, 2017; Richards and Collins, 2020); coach led if–then rules of thumb (heuristics; McPherson, 1999; McPherson and Vickers, 2004) and the development of individual and collective roles and responsibilities (McKay and O’Connor, 2018) through tactical rules (McKay and O’Connor, 2018). Subsequently, we agree with Richards et al. (2012, 2017) position that players should be encouraged to explicitly connect their perception of information to their declarative knowledge-of the game through slow and deliberate environments to make better informed decisions in rapid competitive environments (Ashford et al., 2021).

In contrast, the game presents player’s with incidents where tactical intentions can no longer be referenced i.e., when players have limited time or when game information demands a change in expectations (Passos et al., 2008; McKay and O’Connor, 2018). To support the betterment of player decision making in these moments, coaches can utilise incidental decision making training (Bar-Eli et al., 2011). This approach conforms to variable design principles such as non-linear pedagogy (Chow, 2013; Correia et al., 2019) in that practice provides frequent exposure to the perception of information in order to identify invariances between the information perceived and the action planned (Bar-Eli et al., 2011). Furthermore, in such uncertain environments, coaches should consider guiding players to adopt ‘take-the-first’ heuristics (Raab and Laborde, 2011; Raab and Gigerenzer, 2015) through exploratory coach behaviours (e.g., divergent questioning; Cushion et al., 2011; O’Connor et al., 2018) and practice manipulation (e.g., rule modifications; Passos et al., 2008; Chow, 2013; Balagué et al., 2019; Ashford et al., 2020).

Following the interpretation of our data, we advise coaches to consistently reinforce the following coaching strategies within their practice. First, is the thoughtful development and reinforcement of a common language, where all player’s (and coaches) possess clarity of accepted verbal, visual and haptic information (Richards et al., 2012, 2017; McKay and O’Connor, 2018). If players are able to conceptualise the meaning of terminologies and how they are operationalised during games and training, coaches may consider co-constructing further development of language with the playing group (Richards et al., 2012, 2017). A common language rests on ensuring clarity and coherence between all involved, as a single term must mean the same thing to one player and it does to everyone else. Secondly, we suggest that an adopted common language should be intertwined with a shared “way of playing” housed under a clear common frame of reference (Tee et al., 2018; Ashford et al., 2021). This does not suggest that coaches must prescribe how players behave, but instead suggests that by imprinting strategy (Balagué et al., 2019) coaches can offer a framework as a scaffold for collective and coordinated decision making so players are still able to search for, communicate, decide and act as the game unfolds (Tee et al., 2018).

Thirdly, “What is tactically desirable must be technically possible” (Launder and Piltz, 2013). Thus, coaches should consistently seek to develop and refine technical and physical capabilities of their players as they afford a wider scope of game information to select from Paterson et al. (2013); Wilson et al. (2018). With this, is a caveat that when supporting player’s development of technique, coaches should not shy away from decoupled, massed, blocked and instructional practices (Williams and Hodges, 2005) but ensure that technique is then refined and recoupled to game information. Finally, the findings of this study demonstrate that player’s individual and collective perceptual strategies, retrieval of memory representations, actions and use of language are consistently underpinned by players deep declarative knowledge-of the game (Anderson, 1982; Johnston and Morrison, 2016). By supporting player’s game intelligence and a deeper understanding of *why*, coaches can support players development of self-awareness, meta-cognitive strategies (Price et al., 2019, 2020); realistic evaluations of their performance (MacNamara et al., 2010a, b); valid assessments of physical and technical capabilities (Ashford et al., 2021); and finally, the ability to consider game context during performance to make informed decisions (Levi and Jackson, 2018).

To conclude, this study has presented a breadth of evidence to suggest that game situations in rugby union impact semi-elite players decision making processes in similar ways. In reference to the thorough exploration of player decision making conducted in this study, research should now extend further than semi-elite playing populations and consider elite participants (i.e., Premiership & International players). Furthermore, study into how to best support players development of decision making must progress further than coach perceptions. Instead, we agree with Morgan et al. (2020) conclusive remarks, that future research with rugby union coaches should employ self-confrontation elicitation interviews to explore how the demands of the game are perceived and captured by the coach and impressed onto players within effective learning environments.

## Data Availability Statement

The raw data supporting the conclusions of this article will be made available by the authors, without undue reservation.

## Ethics Statement

The studies involving human participants were reviewed and approved by the Carnegie Faculty Research Ethics Leeds Beckett University. The patients/participants provided their written informed consent to participate in this study.

## Author Contributions

MA, AA, and JP contributed to the proposed purpose, design, materials and methods of the project, ethical considerations, and data analysis. MA contributed to the recruitment and data collection and wrote the manuscript. AA and JP edited and commented the manuscript. All authors contributed to the article and approved the submitted version.

## Conflict of Interest

The authors declare that the research was conducted in the absence of any commercial or financial relationships that could be construed as a potential conflict of interest.

## Footnotes

1 Five UNI players and two PRO players completed the procedure once, therefore their data was removed from the study.

2 The lineout is a set-piece phase of play, which involves the ball being thrown back into the field after it has left (Sayers, 2005). It must have at least two, but commonly includes four to seven, players from each team standing perpendicular to the sideline, 2 m apart with the first player at least 5 m away. The team in possession requires a player to throw the ball straight down the middle of the two lines, whilst two players’ lift a jumper into the air to catch and receive the ball (Croft et al., 2011; Sayers, 2011, 2005). Opponents may also lift someone to compete for the ball. Initiation of movement is often sparked by a ‘caller’ from the team in possession the ball, who then decides where the ball will be thrown and caught. The scrum is also a set-piece situation, which involves a maximum of eight players from each team (forward) forming three rows that are bound together to compete for the ball; (the front row; *hooker* and *props*; second-row’s and back row; *flankers* and *number eight*; Trewartha et al., 2015). The two sides must then bind together to compete for possession of the ball in a coordinated pushing action (Hendricks et al., 2014; Trewartha et al., 2015). For the team in possession of the ball, the scrum is seen as an opportunity to launch attacks, whilst opponent’s often aim to disrupt or steal the ball back (Trewartha et al., 2015). Physical domination can often result in the penalisation of an opposing team (Hendricks et al., 2014). Finally, offensive and defensive moments of the game are defined by the team in possession of the ball (Tee et al., 2018). Rugby union is a unique sport as when a tackle occurs, a physical contest for possession of the ball follows (Tee et al., 2018). This area of the game is called a *ruck* where an attacking team will support a ball carrying player by securing the ball, before it can be passed away to a player in open space. Conversely, the defence are firstly required to tackle a player, which then provides an opportunity to win the ball back (Tee et al., 2018). Therefore, defensive and offensive situations repeatedly ebb and flow between moments of *open-phase play* and *tackle situations*.
